# Structure of Pigment Metabolic Pathways and Their Contributions to White Tepal Color Formation of Chinese *Narcissus tazetta* var. *chinensis* cv Jinzhanyintai

**DOI:** 10.3390/ijms18091923

**Published:** 2017-09-08

**Authors:** Yujun Ren, Jingwen Yang, Bingguo Lu, Yaping Jiang, Haiyang Chen, Yuwei Hong, Binghua Wu, Ying Miao

**Affiliations:** Center for Molecular Cell and Systems Biology, Fujian Provincial Key Laboratory of Haixia Applied Plant Systems Biology, College of Life Sciences, Fujian Agriculture and Forestry University, Fuzhou 350002, China; ryj@fafu.edu.cn (Y.R.); 2160539002@fafu.edu.cn (J.Y.); lubg@fjnu.edu.cn (B.L.); 2564348374@qq.com (Y.J.); 3165402021@fafu.edu.cn (H.C.); 530515316@qq.com (Y.H.); Binghua.wu@fafu.edu.cn (B.W.)

**Keywords:** Chinese narcissus, metabolite analysis, pigment metabolic pathway, tepal pigmentation, comparative KEGG pathway enrichment

## Abstract

Chinese narcissus (*Narcissus tazetta* var. *chinensis*) is one of the ten traditional flowers in China and a famous bulb flower in the world flower market. However, only white color tepals are formed in mature flowers of the cultivated varieties, which constrains their applicable occasions. Unfortunately, for lack of genome information of narcissus species, the explanation of tepal color formation of Chinese narcissus is still not clear. Concerning no genome information, the application of transcriptome profile to dissect biological phenomena in plants was reported to be effective. As known, pigments are metabolites of related metabolic pathways, which dominantly decide flower color. In this study, transcriptome profile and pigment metabolite analysis methods were used in the most widely cultivated Chinese narcissus “Jinzhanyintai” to discover the structure of pigment metabolic pathways and their contributions to white tepal color formation during flower development and pigmentation processes. By using comparative KEGG pathway enrichment analysis, three pathways related to flavonoid, carotenoid and chlorophyll pigment metabolism showed significant variations. The structure of flavonoids metabolic pathway was depicted, but, due to the lack of *F3ʹ5ʹH* gene; the decreased expression of *C4H*, *CHS* and *ANS* genes; and the high expression of *FLS* gene, the effect of this pathway to synthesize functional anthocyanins in tepals was weak. Similarly, the expression of *DXS*, *MCT* and *PSY* genes in carotenoids synthesis sub-pathway was decreased, while *CCD1*/*CCD4* genes in carotenoids degradation sub-pathway was increased; therefore, the effect of carotenoids metabolic pathway to synthesize adequate color pigments in tepals is restricted. Interestingly, genes in chlorophyll synthesis sub-pathway displayed uniform down-regulated expression, while genes in heme formation and chlorophyll breakdown sub-pathways displayed up-regulated expression, which also indicates negative regulation of chlorophyll formation. Further, content change trends of various color metabolites detected by HPLC in tepals are consistent with the additive gene expression patterns in each pathway. Therefore, all three pathways exhibit negative control of color pigments synthesis in tepals, finally resulting in the formation of white tepals. Interestingly, the content of chlorophyll was more than 10-fold higher than flavonoids and carotenoids metabolites, which indicates that chlorophyll metabolic pathway may play the major role in deciding tepal color formation of Chinese narcissus.

## 1. Introduction

There are many different colored flowers in the world. Color make flowers attractive and facilitates the reproduction of flowering plants. Besides that, colored flowers can also be used by humans to beautify their environment, in addition to the health benefits of pigments as dietary components, which provide protection against a wide range of human diseases [1,2,3]. As a critical index to judge the ornamental and/or economic value, understanding the molecular basis of flower color formation would improve the applicable occasions of flowers. The mechanism of flower color formation has been studied in the past three decades [4]. Conventionally, flower color is an evolutionary result [5,6,7,8,9,10], and in most cases thought to be decided by three classes of pigments: flavonoids, carotenoids and alkaloids [4,11]. Flavonoids are the collective name of multiple secondary metabolites originated from 2-benzene chromone core, which are further classified into six major groups, chalcones, flavones, flavonols, flavandiols, anthocyanins, and condensed tannins (or proanthocyanidins), that exist in most higher plants [12,13]. Among them, anthocyanins, as the chief flavonoid pigments, confer a diverse range of color from orange, red to violet and blue. Other flavonoids such as flavones and flavonols cover the range from ivory white to pale yellow or invisible to human beings, therefore act as accessory pigments [11]. Carotenoids are the general name of carotenes and phytoxanthins, most of which give flowers yellow, orange or red color [11]. Alkaloids contain three types of pigments: βines, papaverines and berberines. Βines make flowers appear red or yellow color, papaverines make flowers appear yellow color, while berberines make flowers appear orange or yellow color [14]. Actually, besides the above pigments, in some plants or other species of chrysanthemum, rose, peony and orchid, chlorophyll also acts as a color pigment in the green flower variants [15,16]. Meanwhile, studies also discovered that the concentration of pigments [17], the mixed effect of diverse pigments [18], the chelation effect of pigments with metal ions [19,20], the pH value of vacuoles [21], and other physical (such as light, temperature and humidity) or chemical (such as vacuolar ion species) factors also have effects on the output of flower color [22]; nevertheless, the composition of pigments in flower is the most important determinant [22]. In recent years, some color-associated structural or regulatory genes involved in flower color formation have been identified; among them, some genes such as *F3ʹ5ʹH*, *CHS*, *F3ʹH*, *DFR*, *ANS* and *PSY* have already been applied in genetic engineering to create new flower colors [23,24,25,26,27,28,29]. These work provide valuable reference for color innovations of other flowering plants. Nevertheless, white color can be the result of various factors [30,31], and the systematic explanation of white flower formation in plants is still fragmentary, therefore the color innovation of this kind of plants is still difficult.

Chinese narcissus (*Narcissus tazetta* var. *chinensis*) belongs to perennial herb of Amaryllidaceae. It is one of the ten traditional flowers in China and a famous bulb flower in the world flower market. There are two major cultivated varieties of Chinese narcissus, one is called “Jinzhanyintai”, its flower has six petal-like tepals surmounted by a cup-shaped corona, and the color of tepals are white while the corona is yellow. Another variety is called “Yulinglong”, it has 12 petal-like white tepals rolled into a cluster with no obvious corona, and its yellow stamens specialized into petal-like structures. Besides the flower type, Jinzhanyintai is more fragrant than Yulinglong, hence it is the most widely cultivated variety. Favoring for the beautiful flower pattern and sweet fragrance, Chinese narcissus has high ornamental and economic values [23]. However, the cultivated species only have white tepal, which largely limits their applicable occasions. The modification of tepal color may help to solve this problem.

Unfortunately, due to the lack of genome information of narcissus species, the molecular basis of tepal color formation of Chinese narcissus has not been established. Most prior studies were focused on the identification of single pigment-related gene [24] or the composition of flavonoids and carotenoids in narcissus cultivars and their relationship with flower color [18,32], but no systematic study on the molecular structure of pigment metabolic pathways exist in tepal and their contributions to pigments synthesis and degradation and their influences on tepal color formation. Luckily, as the strategy using transcriptome profile with no genome reference to dissect biological phenomena was proved to be effective [25], the above aim to explain white color tepal formation in Chinese narcissus becomes possible. To achieve this goal, in this study, transcriptome profile and pigment metabolite analysis methods were used in tepals at different flower development and pigmentation stages to discover the pigment metabolic pathways and critical structural genes that are involved in white tepal formation in the most cultivated variety “Jingzhanyintai”. This work provides valuable theoretical basis for Chinese narcissus flower color innovations in future.

## 2. Results

### 2.1. Tepal Pigmentation Characteristics of Chinese Narcissus

**Figure 1:**
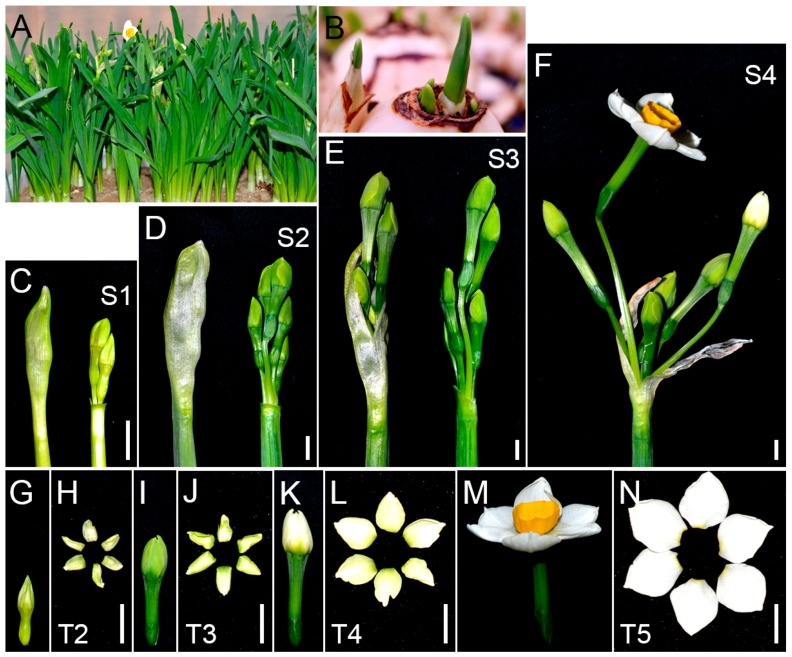
Flower development and tepal pigmentation characteristics of Chinese narcissus cultivated variety “Jinzhanyintai”. (**A**) An overview of flower development of “Jinzhanyintai” after cultivated for 20 days in greenhouse. The sheathed leaves are robust and some flowers are opening; (**B**) Shoots with sheathed leaves come out from bulbs after planting for five days. This is the first time point for collecting tepals for RNA-seq (S1,T2); (**C**,**G**,**H**) Spathe with wrapped umbel (flower buds) was isolated from cultured bulbs five days after planting. The whole spathe and flower buds were completely embedded in shoots and displayed faint-yellow color (**C**,**G**); Tepals (T2) were collected and used for RNA-seq and metabolites analysis (**H**); (**D**,**I**,**J**) Spathe with wrapped umbel (flower buds) was isolated from bulbs 10 days after planting. Unopened spathes with wrapped umbel have emerged out from bulbs and turned deep-green (**D**,**I**); This was the second time point for collecting tepals for RNA-seq and metabolites analysis (S2,T3) (**J**); (**E**) New splited spathe with deep-green umbel (flower buds) isolated from bulbs 16 days after planting. The color of tepals at this time point (S3) was the same as in tepals at S2; (**F**) Umbel with opening flower and flower buds close to open after planting for 19 days. The color of tepals at this time point (S4) will gradually fade, turn from deep-green to green/white, and at last turn into pure white in opening flowers; (**K**,**L**) Green-to-white transition buds (**K**) and tepals (**L**). Tepals at this stage (T4) were collected and used for RNA-seq and metabolites analysis; (**M**,**N**) Opening Chinese narcissus flower (**M**) and its pure white tepals (**N**). Tepals at this stage (T5) were collected for RNA-seq and metabolites analysis. Bar = 1 cm.

Flower development and tepal pigmentation characteristics of Chinese narcissus were observed in the most widely cultivated variety “Jingzhanyintai” (Figure 1). Flowers initiate in bulbs during summer dormancy [23]. The newly formed flower buds are nearly colorless. After five days of planting (DAP), bulbs grow shoots with sheathed leaves (Figure 1B). At this time point (Figure S1), spathes stay in deep center of shoots with wrapped umbel, and the inside tepals display faint-yellow color (Figure 1C,G,H). At 10 DAP, unopened spathes with wrapped umbel grow bigger and emerge out from bulbs (Figure 1D). At this time point (S2), the flower buds and their tepals in spathe turn into deep-green (Figure 1I,J). At time point S3 (16 DAP), spathes split and umbel with flower buds come out (Figure 1E). After spathes are completely dehiscent (S4, 19 DAP) (Figure 1F), the color of tepals gradually fades, turning from deep-green to white (Figure 1K,L). After that, the flower buds open and the tepal color in mature flowers turn into pure white (Figure 1M,N). During this whole process (about 20 days), the tepal color experiences five typical stages: colorless (T1), faint-yellow (T2), deep-green (T3), green-to-white (T4) and pure white (T5) (Figure 1). As T2 to T5 stages show the most significant tepal color changes, they should cover the most important regulations that result in white mature tepals; therefore, tepals at these four color stages were collected for subsequent RNA-seq and pigment metabolites analysis.

### 2.2. Tepal Transcriptome Sequeincing, Assembly and Annotation

To gain the tepal transcriptome of Chinese narcissus, one cDNA library (Tmix) for RNA-seq (equally mixed RNA from T2, T3, T4 and T5 tepals) and another four cDNA libraries for digital gene expression profiling (DGE) analysis (RNA from separate T2, T3, T4 and T5 tepals) were created and sequenced. About 34.7 million 101-bp paired clean reads were obtained in the RNA-seq library, and an average of six million paired reads were obtained in each DGE library (1). After using Trinity platform to de novo assemble the tepal transcriptome, all reads from both the RNA-seq and the four DGE-seq libraries were assembled into 3,302,699 contigs with a mean length of 64 bp, these contigs were further assembled into 134,985 transcripts with a mean length of 1018 bp, and obtained 63,141 unigenes with a mean length of 704 bp (Table S2). The reads in each library could reach a coverage more than 80% of the assembled transcriptome (Table S3), their expression have achieved saturation, and their sequences can equally distribute on the assembled uni-transcripts (Figure S2). These results indicate that the assembled tepal transcriptome has high quality; it is sufficient to reflect the composition and expression of unigenes in tepals.

**Figure 2:**
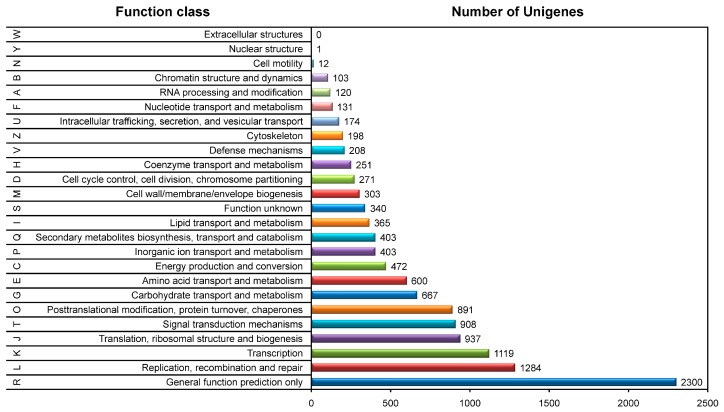
Summary of COG function annotation of assembled tepal transcriptome of Chinese narcissus. In total, 8977 annotated transcripts were assigned to 25 eukaryotic orthologous groups based on sequence similarity. The number of transcripts in each group was labeled.

To make annotation of the assembled tepal transcriptome, all of the 63,141 unigenes were aligned against Nr (NCBI non-redundant database), Swiss-Prot, COG (Cluster of Orthologous Groups of proteins), GO (Gene Ontology) and KEGG (Kyoto Encyclopedia of Genes and Genomes) databases by using BLASTx. In total, 29,730 unigenes provide significant hits (Table S4). Among them, 8977 unigenes were annotated in COG database based on sequence similarity. Except for the function group “General function prediction only”, the groups “Replication”, “Transcription”, “Translation”, “Signal transduction”, and “Posttranslational modification” share higher frequency of unigenes among the COG groups, then followed by “Carbohydrate”, “Amino acid”, “Inorganic ion” and “Lipid” related transport and metabolism groups, the “Secondary metabolites biosynthesis, transport and catabolism” and the “Energy production and conversion” groups (Figure 2). This indicates that the annotated unigenes have various functions in tepals, especially in DNA replication, transcription, translation, posttranslational modification, and substance and energy metabolisms.

**Figure 3:**
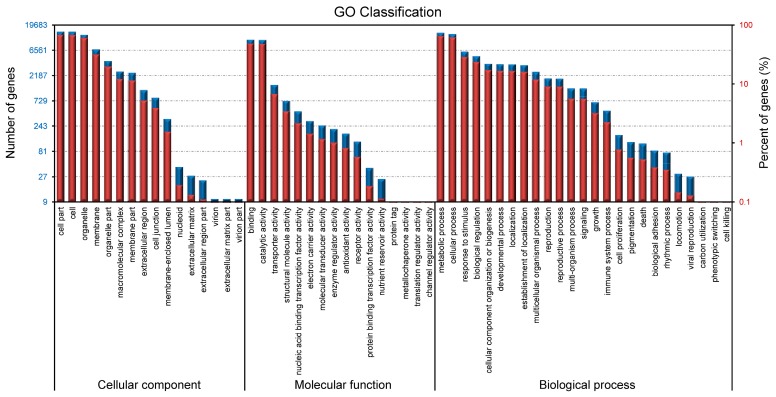
Summary of GO function annotation of assembled tepal transcriptome of Chinese narcissus. The composition of the second level GO terms is divided into three major categories: biological process, cellular components, and molecular function. The terms in each category are sorted based on the number of unigenes in each term. Percentages of unigenes in each term of the three categories are shown.

To further annotate the tepal transcriptome by GO, 21,368 unigenes with significant hits were classified into three Gene Ontology categories: biological process, molecular function, and cellular components (GO level 2) (Figure 3 and Table S4). In cellular component category, “Cell part” and “cell” groups had the highest ratio of unigenes. In molecular function category, a high percentage of unigenes fell into the “binding” and “catalytic activity” groups. While in biological process category, the most abundant unigenes were assigned to “metabolic process” and “cellular process” groups. Remarkably, 13,921 unigenes were classified into “metabolic process” group, which indicates that a considerable number of GO annotated unigenes may be involved in diverse primary and secondary metabolic processes in tepals.

In total, 6801 unigenes were assigned to 135 pathways annotated by KEGG classification system (Table S5). Among them, the majority of the identified enzymes, which exist in various essential pathways, are conserved in different plant species, indicating that the assembled tepal transcriptome in this research is reasonably complete. By using “Overview and global mapping” strategy, 2019 unigenes were assigned to “metabolic pathways” and 898 unigenes were assigned to “biosynthesis of secondary metabolites” (Table S6). These unigenes provide resources for identifying specific pathways and genes that may involve in tepal development and pigmentation of Chinese narcissus.

### 2.3. Metabolic Pathway Enrichment in Tepals During Tepal Development and Pigmentation Processes

**Figure 4:**
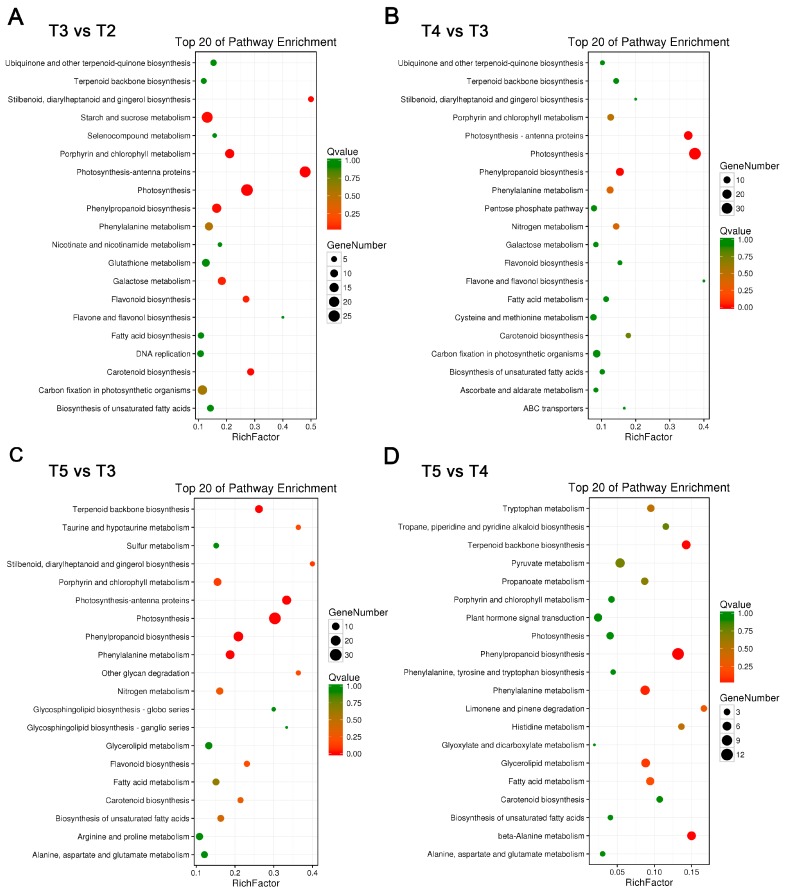
Enrichment of metabolic pathways at different tepal development and pigmentation stages. (**A**–**D**) Statistics of pathway enrichment in: T3 vs. T2 (**A**); T4 vs. T3 (**B**); T5 vs. T3 (**C**); and T5 vs. T4 (**D**) tepal transcriptome comparisons. The top 20 enriched pathways based on q-values in each comparison and the number of unigenes in each pathway are shown.

As described above, the color of tepals from T2 to T5 stage showed significant differences (Figure 1), which indicates considerable changes of pigment associated metabolic pathways during tepal development and pigmentation process. To make this clear, the enrichment of metabolic pathways in the four sequenced tepal DGE libraries from T2 to T5 stages were compared. In T3 versus T2 comparison, the top 20 enriched metabolic pathways were excavated (Figure 4A), which can be classified into four major classes: photosynthesis, flavonoids metabolism, carbohydrate metabolism, and carotenoid biosynthesis. Among them, the photosynthesis class contains “antenna proteins synthesis”, “photosynthesis”, and “porphyrin and chlorophyll metabolism” branches sorted by q-value; the flavonoids metabolism class contained branches listed as “phenylpropanoid biosynthesis”, “stilbenoid, diarylheptanoid and gingerol biosynthesis”, and “flavonoid biosynthesis”, with the front two are involved in precursor biosynthesis of diverse flavonoid metabolites [26]; and the carbohydrate metabolism class contains branches sorted by “starch and sucrose metabolism”, “galactose metabolism”, and “carbon fixation in photosynthetic organisms”, which display dynamic material synthesis and accumulation during the early rapid tepal development stage. In T4 versus T3 comparison, the top 20 enriched metabolic pathways were concentrated in photosynthesis, flavonoids metabolism, nitrogen metabolism, and carotenoid biosynthesis classes, and showed notable difference from the early rapid tepal development stages (Figure 4B). The branches in photosynthesis class are changed into the sorting order “photosynthesis”, “antenna proteins synthesis”, and “porphyrin and chlorophyll metabolism”. The branch items “phenylpropanoid biosynthesis” and “phenylalanine metabolism” are ranked as the top two in the flavonoids metabolism class, though the “flavone and flavonol biosynthesis” and “flavonoid biosynthesis” items also show enrichment. Nitrogen metabolism showed perceptible enrichment in T4 versus T3 comparison, which indicates vibrant activities of amino acid, nucleotide, and protein biosynthesis related to nitrogen balance to control the growing speed of tepals. In T5 versus T3 comparison, besides the general enrichment of the four major metabolism classes in T4 versus T3 comparison, more classes related to organic acid metabolism (“taurine and hypotaurine metabolism”, “biosynthesis of unsaturated fatty acids”, and “fatty acid metabolism”), lipid and glycan metabolism (“glycerolipid metabolism” and “other glycan degradation”), and amino acid metabolism (“arginine and proline metabolism” and “alanine, aspartate and glutamate metabolism”) were emerged (Figure 4C). In T5 versus T4 comparison, enriched pathways were mainly clustered in the classes of flavonoids metabolism (“phenylpropanoid biosynthesis” and “phenylalanine metabolism”), various amino acids metabolism (“β-alanine metabolism” is the dominant), carotenoids metabolism (“terpenoid backbone biosynthesis” is the dominant), organic acids metabolism (glycerolipid, fatty acid, and pyruvate metabolisms), aroma metabolism (limonene and pinene degradation), and alkaloid biosynthesis (tropane, piperidine and pyridine alkaloid biosynthesis) (Figure 4D), which showed significant differences from the front three comparisons. The T5 versus T4 comparison highlighted the transition from color pigments metabolism to aroma metabolism to form fragrance in tepals during this interval. Therefore, according to the above four comparisons, it showed that the conventional color-related flavonoids and carotenoids metabolic pathways are obviously involved in tepal development and pigmentation regulations. Additionally, for chlorophyll metabolic pathway showed significant enrichment in T3 versus T2 and T4 versus T3 comparisons, the involvement of chlorophyll metabolism in tepal pigmentation regulations should be also regarded as important. Thus, these three pigment metabolic pathways were chosen for further analysis.

### 2.4. Structure of Flavonoids Metabolic Pathway, the Gene Expression Variation, and Content Changes of Flavonoid Metabolites in Tepals during Tepal Pigmentation Process

**Figure 5:**
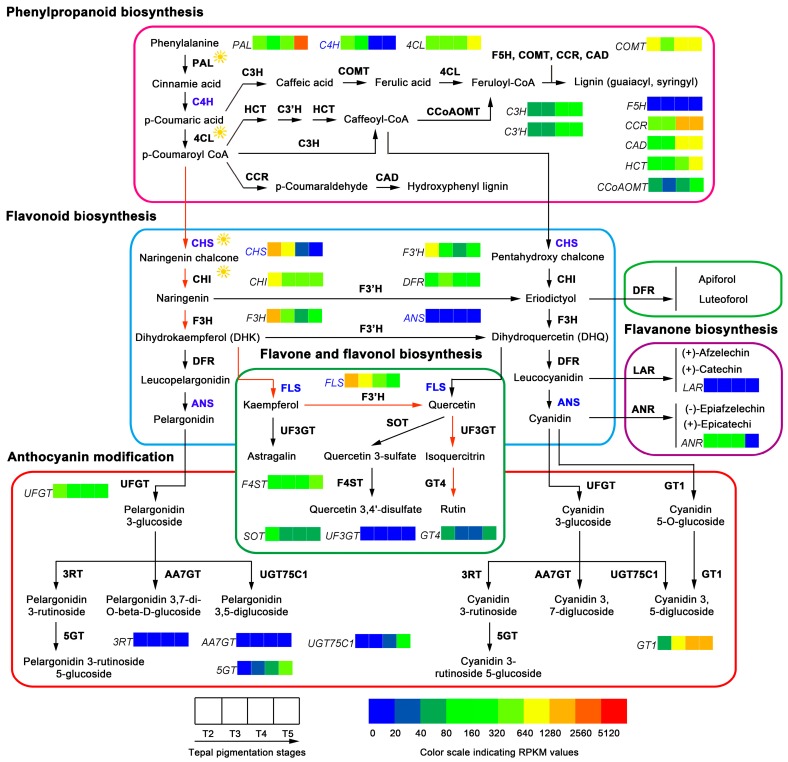
Flavonoids metabolic pathway in tepals of Chinese narcissus and gene expression changes during tepal pigmentation process. The pathway is divided into five sub-pathways based on the functions of unigenes. The additive expression changes of unigenes at different tepal pigmentation stages (T2, T3, T4 and T5) are shown by color grids based on RPKM value. The presumed critical genes *C4H*, *CHS*, *ANS*, and *FLS* in the pathway are marked with blue color. Black filled arrows indicate the substance metabolism directions; red filled arrows indicate the main flux (from naringenin to rutin) of flavonoids metabolic pathway in tepals. Enzymes marked with sunlight indicate their catalytic activities can be influenced by light.

Genes encoding the members of flavonoids metabolic pathway in tepals were identified using the assembled tepal transcriptome. They were searched based on standard enzyme names and synonyms in combined function annotations. By mapping to the KEGG reference pathway, 114 unigenes encoding 33 enzymes have been identified (Table 1 and Table S7). Among them, C4H, C3H/C3′H, F5H, ANS, 3RT, AA7GT, LuOMT, F4ST, UF3GT, ANR and LAR enzymes only correspond to one unigene, but most of the other enzymes such as 4CL, CCR, CAD, HCT, CHS, UFGT and 3′GT correspond to two or more unigenes, which belong to multiple gene families (Table S8). Remarkably, the genes encoding C2H, F3′5′H, FNS, AS1, and IFS enzymes and many of the other genes related to anthocyanin glycosylation (*3GGT*, *UGT79B1*, *3AT*, *5AT*, and *UGAT*) and flavonoids modifications (e.g., acetylation, methylation, and sulfonylation) (*Mt1/Mt2*, *Mf1/Mf2*, *3MaT1*, *3MaT2*, *5MaT2*, and *UA3′5′GZ*) could not find corresponding unigene (Table S9). The F3′5′H enzyme is essential for creating blue pigments through the delphinidin-based anthocyanins synthetic sub-pathway [27,28]. The lack of *FNS*, *IFS* and *AS1* unigenes indicates that apigenin (a flavone), 2-hydroxyisoflavanone and aurone metabolites could not be produced in tepals [12,29]. Based on the above-identified unigenes, the proposed flavonoids metabolic pathway in tepals of Chinese narcissus was constructed (Figure 5). The pathway was divided into five sub-pathways according to the functions of each sub-pathway that was previously described in other studies [12,13,29,30].

**Figure 6:**
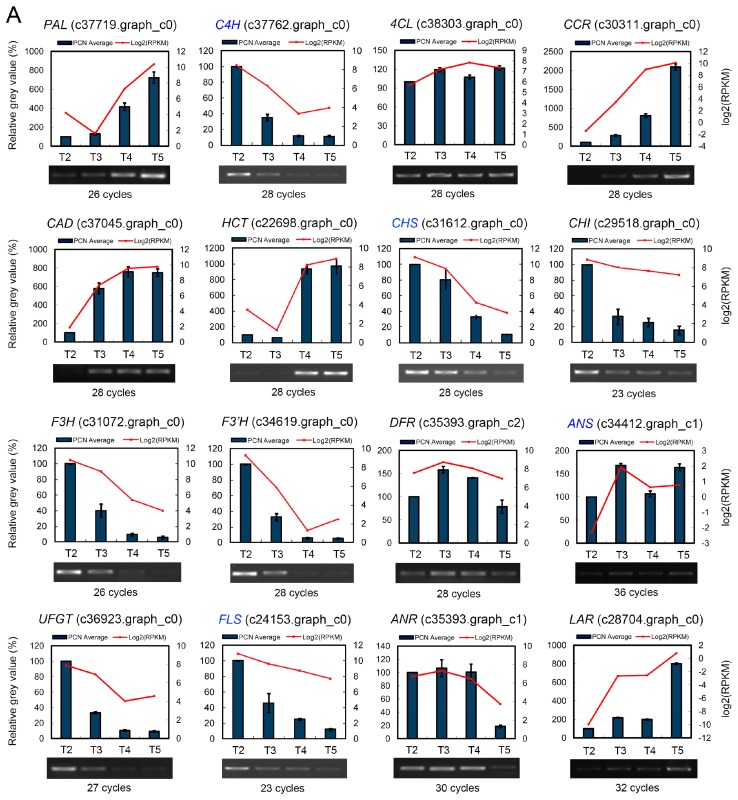

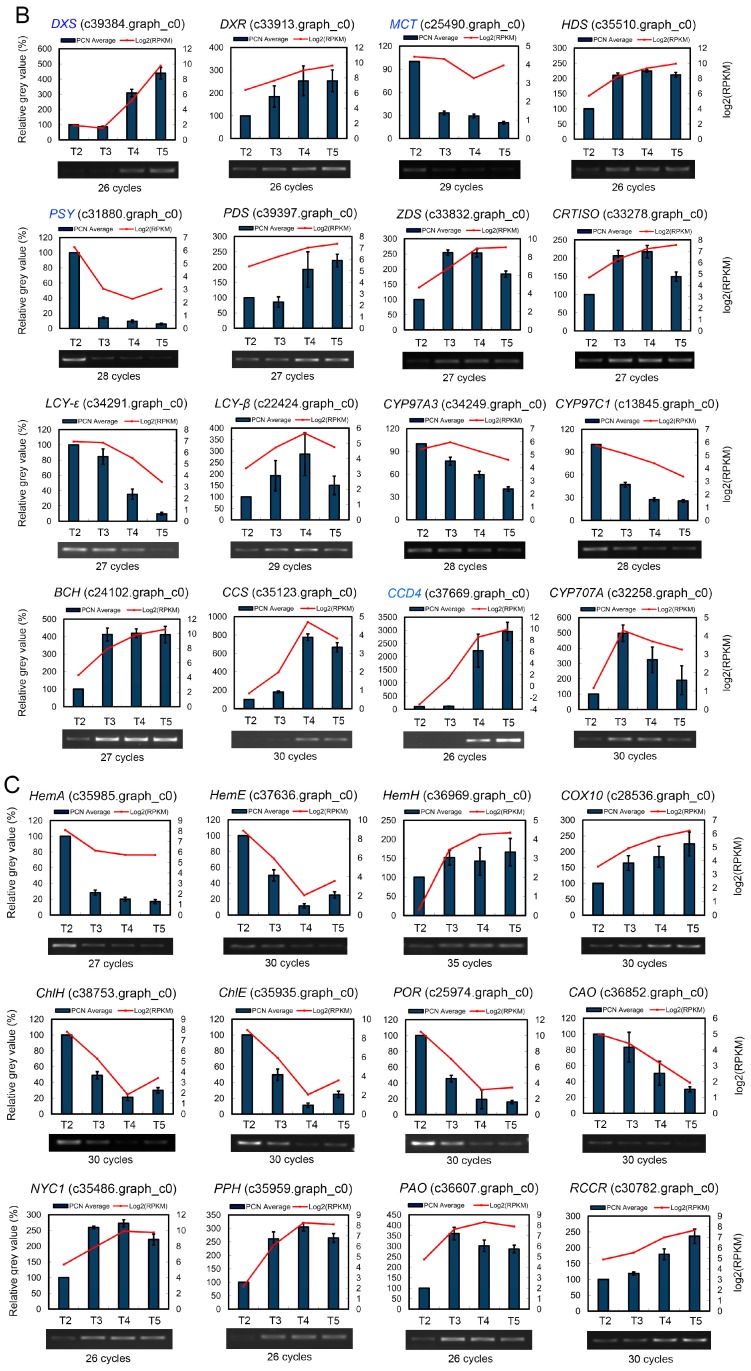
Semi-qRT-PCR validation of unigene expression patterns in flavonoids, carotenoids and chlorophyll metabolic pathways in tepals during pigmentation process: (**A**) expression of 16 unigenes of flavonoids metabolic pathway; (**B**) expression of 16 unigenes of carotenoids metabolic pathway; and (**C**) expression of 12 unigenes of chlorophyll metabolic pathway. Expression of all unigenes was confirmed in three biological repetitions with three technical replications. PCR cycles used to amplify specific genes were marked. The expression strength for each unigene was converted into grey value by software AlphaView. Relative expression level during four tepal pigmentation stages were recalculated after setting the grey value at T2 as 100%. Expression change trends of unigenes detected by semi-qRT-PCR were compared with the result of DGE-seq represented by RPKM values. The unigene *GAPDH* (*c28660.graph_c0*) was used as an inner control.

The expression change trends of the 114 unigenes during tepal pigmentation process based on RPKM value were compared. The expression strength of all the multiple enzyme family genes was represented by the additive effect of all the unigenes in the same family, supposed that every unigene contributes to the enzyme activity. Briefly, the genes encoding 11 enzymes, PAL, 4CL, C3H/C3′H, CCR, CAD, HCT, CCoAOMT, 5GT, UGT75C1, GT1 and F4ST, showed increased expression trends in tepals from T2 to T5 stages, while genes encoding another 11 enzymes, C4H, CHS, CHI, F3H, F3′H, UFGT, 5MaT1, 3′GT, FLS, SOT and ANR, showed overall decreased expression, and the genes encoding the remaining enzymes, COMT, F5H, DFR, ANS, 3RT, AA7GT, LuOMT, GT4, UF3GT and LAR, showed nearly parallel expression. The single transcript unigene corresponding to ANS, 3RT, AA7GT, UF3GT and LAR enzyme respectively showed extremely low expression level throughout the whole tepal pigmentation process (Figure 5). The sharply decreased expression of *C4H*, *CHS*, *F3H*, *F3′H* and *UFGT* plus the extremely low expression of *ANS*, *3RT* and *AA7GT* unigenes indicates that anthocyanins formation (red, purple and blue pigments) in tepals was negatively regulated, not only in the precursor synthesis step (controlled by *C4H* and *CHS*), but also in the anthocyanidins synthesis and modification steps (controlled by *F3H*, *F3′H*, *ANS*, *UFGT*, *3RT* and *AA7GT*). Additionally, the increased expression of *C3H/C3′H*, *CCR*, *CAD*, *HCT*, *CCoAOMT* and the high expression of *FLS* unigenes in other flavonoids metabolic sub-pathways also showed competition to the anthocyanins biosynthesis sub-pathway (Figure 5). To confirm the gene expression profile of DGE-seq dataset, the expression patterns of representative *PAL*, *C4H*, *4CL, CCR, CAD, HCT*, *CHS*, *CHI*, *F3H*, *F3′H*, *DFR*, *ANS*, *UFGT*, *FLS*, *ANR* and *LAR* unigenes were analyzed by semi-qRT-PCR in tepals during the tepal pigmentation process (Figure 6A). The results showed that all expression change trends of the detected unigenes were consi stent with the DGE-seq result, which proved the overall effect of flavonoids metabolic pathway that negatively controls anthocyanins formation in tepals on transcription level.

**Figure 7:**
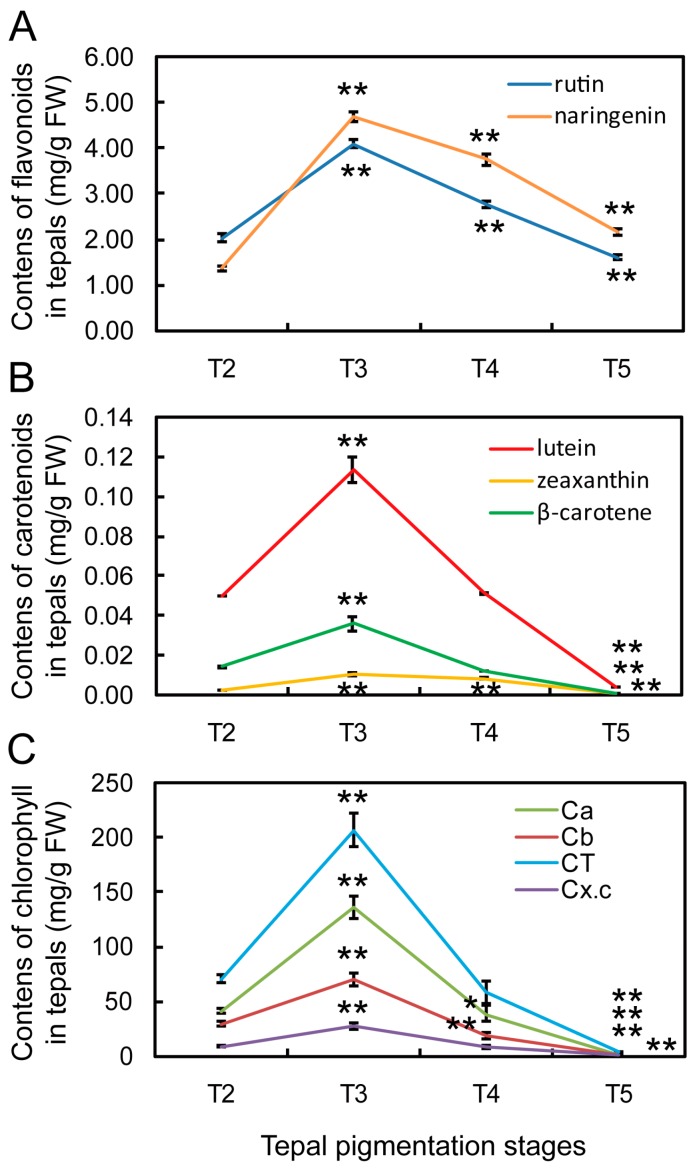
Content changes of flavonoid, carotenoid and chlorophyll metabolites in tepals of Chinese narcissus during tepal pigmentation process: (**A**) rutin and naringenin in flavonoids metabolic pathway; (**B**) β-carotene, lutein and zeaxanthin in carotenoids metabolic pathway; and (**C**) chlorophyll a (Ca), chlorophyll b (Cb), total chlorophyll (CT) in chlorophyll metabolic pathway and the total carotenoids (Cx.c) in carotenoids metabolic pathway. All detections were carried out in at least three independent biological repetitions with three technical replications. The results were displayed as mean value ± SE (standard error). FW, fresh weight. Significant differences between T2 and other stages were made by software Origin 7.5 using student’s *t*-tests. ** represents *p* < 0.01, * represents *p* < 0.05.

To further make clear the correlation of gene differential expression with content changes of flavonoid metabolites in tepals, seven intermediate metabolites (caffeic acid, naringenin, eriodictyol, DHQ, quercetin, kaempferol, and rutin) in flavonoids metabolic pathway were detected by HPLC (Figure S3A,B). The results showed that naringenin and rutin are the main flavonoid metabolites in tepals, while the others are too low to be detected (Table S10). The contents of naringenin and rutin at different tepal pigmentation stages were compared and showed that both were increased from T2 to T3 stage, achieved to peak at T3 stage, and then gradually decreased at T4 and T5 stages (Figure 7A). The change extent of naringenin was higher than rutin in most stages, which was consistent with the cognition that naringenin is a precursor of rutin through the F3H-FLS-F3′H-UF3GT-GT4 sub-pathway (Figure 5), in which the FLS enzyme play a critical role.

### 2.5. Structure of Carotenoids Metabolic Pathway, the Gene Expression Variation, and Content Changes of Carotenoid Metabolites in Tepals During Tepal Pigmentation Process

**Figure 8:**
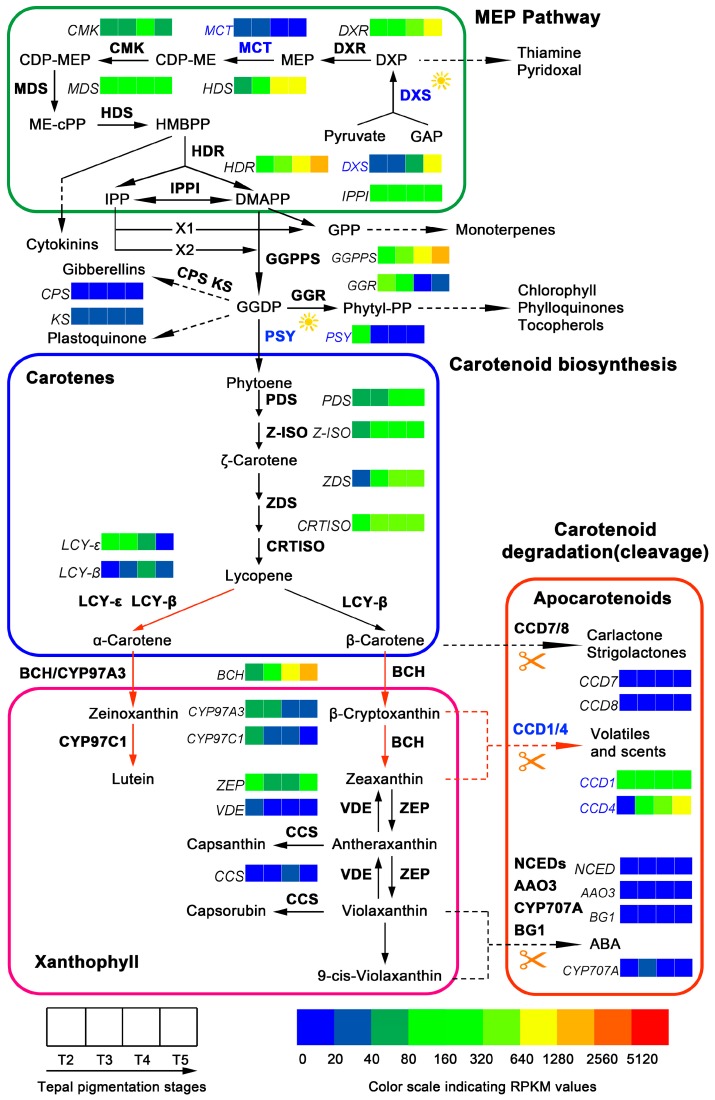
Carotenoids metabolic pathway in tepals of Chinese narcissus and gene expression changes during tepal pigmentation process. The pathway is divided into three sub-pathways based on the functions of unigenes. The additive expression changes of unigenes at different tepal pigmentation stages (T2, T3, T4 and T5) are shown by color grids based on RPKM value. The presumed critical genes *DXS*, *MCT*, *PSY*, and *CCD1/CCD4* in the pathway are marked with blue color. Black filled arrows indicate the substance metabolism directions; red filled arrows indicate two dominant flux of carotenoids metabolic pathway in tepals. Enzymes marked with sunlight indicate their catalytic activities can be influenced by light.

Genes encoding the members of carotenoids metabolic pathway in tepals of Chinese narcissus were analyzed. By mapping to the KEGG reference pathway, 61 unigenes corresponding to 30 enzymes were identified in the tepal transcriptome. Among them, 14 unigenes belong to carotene precursor synthesis step (MEP pathway), 31 unigenes belong to carotenoids synthesis step, and 16 unigenes belong to carotenoids degradation/cleavage step (Table 2 and Table S11). DXR, MCT, CMK, MDS, HDR, Z-ISO, ZDS, LCY-ε, LCY-β, CYP97A3, CYP97C1, CCS, CCD8, CCD7, NCED1 and AAO3 enzymes only correspond to one unigene each, while DXS, HDS, GGPPS, PSY, PDS, CRTISO, BCH, ZEP, VDE, CCD1, CCD4, CYP707A and BG1 enzymes correspond to two or more unigenes (Table S12). Notably, some enzymes, such as NSY, CrtO, CrtW, ABA2 and AOG, in the classic carotenoids metabolic pathway could not find corresponding unigene(s) (Table S13). The lack of CrtO, CrtW and NSY indicates that carotenoids metabolic pathway at β-carotene node can only go through the BCH-ZEP branch to synthesize zeaxanthin, violaxanthin and abscisic acid (ABA), and/or catalyzed by CCD enzymes to generate strigolactone, carlactone, volatiles and scents [33,34]. Besides the main carotenoids metabolic pathway, several branches related to tocopherols, phylloquinones, chlorophyll, monoterpenes, plastoquinone, gibberellins, cytokinins, thiamine and pyridoxal synthesis were also analyzed, and three unigenes, *CPS*, *KS* and *GGR*, were identified (Table S12). Based on these identified unigenes, the proposed carotenoids metabolic pathway in tepals of Chinese narcissus was constructed (Figure 8).

The expression change trends of the 61 unigenes and the three branched pathway genes in tepals during pigmentation process were compared based on their RPKM value. The expression level of the multiple enzyme family unigenes was calculated according to the method described in above part. Briefly, in carotenoids precursor synthesis step (MEP pathway), the first gene *DXS* and the third gene *MCT* showed extremely low expression at T2 and T3 stages, then *DXS* was increased and achieved its top level at T5 stage, but *MCT* was further decreased to an even lower level. Other genes such as *DXR*, *HDS* and *HDR* showed firstly moderate but then increased expression, but the genes *CMK*, *MDS* and *IPPI* showed moderate and nearly parallel expression (Figure 8). In carotenoids biosynthetic step, *GGPPS*, *PDS*, *Z-ISO*, *ZDS*, *CRTISO* and *BCH* genes showed significantly increased expression, whereas *PSY*, *LCY-ε*, *CYP97A3* and *CYP97C1* genes showed decreased expression. Remarkably, the *PSY* gene was sharply decreased to an extremely low level from T3 stage. *LCY-β* and *CCS* genes showed firstly increased but then decreased expression, and the level of *CCS* and *VDE* were extremely low. *ZEP* gene showed moderate expression that was firstly decreased at T3 and T4 stages but then increased to its original level at T5 stage. The expression of *LCY-ε* was much higher than *LCY-β* at T2 and T3 stages, but after T4 stage its expression was decreased and close to the level of *LCY-β*, which indicates that the *α*-carotene sub-pathway activity should be higher than the *β-carotene* sub-pathway before T4 stage. In carotenoids degradation/cleavage step, nearly all of the genes in NCEDs-ABA branch (*NCED1*, *AAO3*, *CYP707A* and *BG1*) were constantly expressed at extremely low level, but in other degradation/cleavage sub-pathways the expression of four *CCD* genes displayed notable difference. The expression of *CCD1* was moderate and steady during the whole tepal pigmentation process, while *CCD4* showed significant increase, and at T4 and T5 stages its expression was much higher than *CCD1*. Contrarily, the expression of *CCD7* and *CCD8* genes always showed extremely low level (Figure 8). These results indicate that *CCD1* and *CCD4* should play the major role in carotenoids degradation/cleavage sub-pathway. Coincidently, both of them have been proven to negatively regulate carotenoid accumulation in plants [31,32,33,34,35,36,37,38,39,40]. To assure the gene expression profile of DGE-seq, 16 representative unigenes were selected for expression verification by semi-qRT-PCR experiments. The results showed that all expression change trends of the detected unigenes could well match to the DGE-seq result (Figure 6B), which confirmed the overall negative effects of carotenoids metabolic pathway on the synthesis of color carotenoid pigments in tepals on transcription level.

Previous studies have discovered that β-carotene, lutein, zeaxanthin and astaxanthin are the major flower pigments in carotenoids metabolic pathway [11,41]. Hence, the existence and contents of these four metabolites were detected by HPLC in tepals (Figure S4A,B). The results showed that lutein is the most abundant carotenoid metabolite whenever in any stage, then followed by β-carotene and zeaxanthin, but astaxanthin was not detected (Table S14). By comparing content change trends of the three metabolites at different tepal pigmentation stages, all of them were peak at T3 stage, but then continuously decreased to extremely low level at T5 stage, which displayed similar change tendency (Figure 7B). Nevertheless, no matter which metabolite, their content in tepals was still very low (Figure 7B and Table S14). This may be the reason why tepals do not put on yellow color like corona (lutein content in corona was about 100-fold higher than in tepals at T4 and T5 stages (data not shown) in Chinese narcissus (Figure 1).

### 2.6. Structure of Chlorophyll Metabolic Pathway, the Gene Expression Variation, and Content Changes of Chlorophyll Metabolites in Tepals During Tepal Pigmentation Process

**Figure 9:**
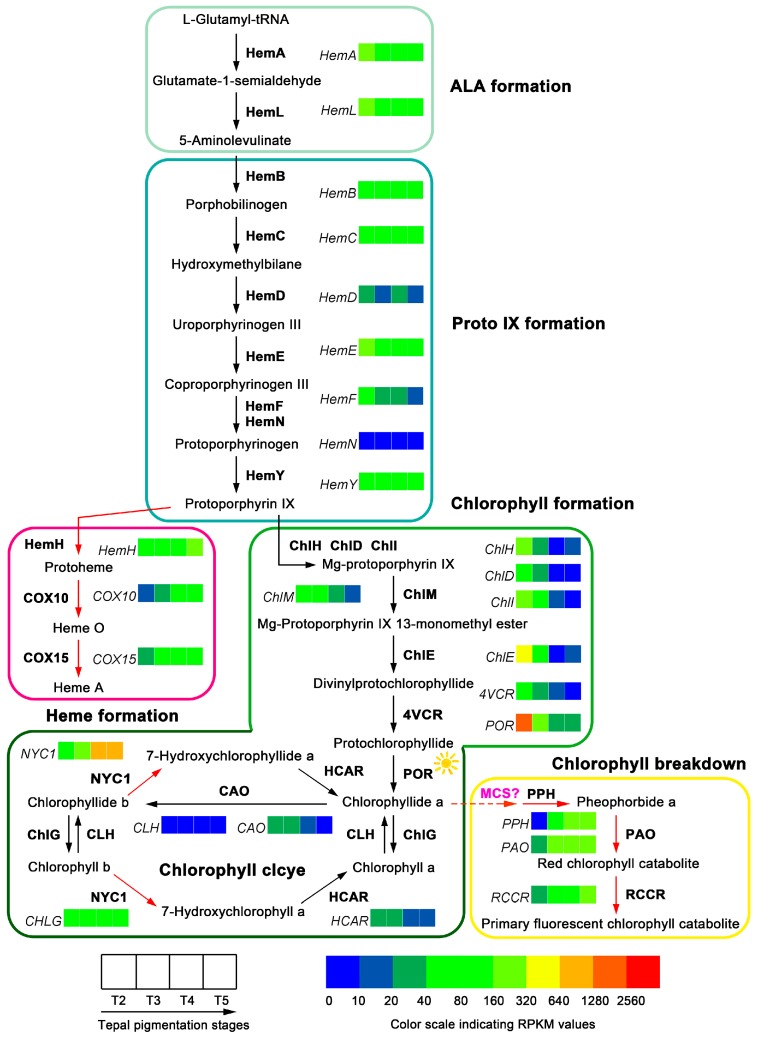
Chlorophyll metabolic pathway in tepals of Chinese narcissus and gene expression changes during tepal pigmentation process. The pathway is divided into five associated parts (ALA formation, Proto IX formation, heme formation, chlorophyll formation, and chlorophyll breakdown) based on the functions of unigenes. The additive expression changes of unigenes at different tepal pigmentation stages (T2, T3, T4 and T5) are shown by color grids based on RPKM value. Black filled arrows indicate the substance metabolism directions; red filled arrows indicate two dominant flux of chlorophyll metabolic pathway in tepals. Enzymes marked with sunlight indicate their catalytic activities can be influenced by light. Interaction of HCAR with other components in chlorophyll metabolic pathway has not been investigated, the molecular nature of MCS is unknown, thus are labeled with a question mark.

Fifty-one unigenes corresponding to 27 enzymes of the classic chlorophyll metabolic pathway were identified after mapping the tepal transcripome to the KEGG reference pathway (Table 3 and Table S15). In ALA, Proto IX, heme and chlorophyll biosynthesis steps [42], enzymes like HemB, HemD, HemN, COX10, ChlD, ChlM, ChlE, 4VCR, ChlG and CAO can find one corresponding unigene, while others such as HemA, HemL, HemC, HemE, HemF, HemY, HemH, COX15, ChlH, ChlI, and POR can find two or more corresponding unigenes (Table S16). The enzymes like HemG, BchE, BchJ, ChlL, ChlN and ChlB in the classic pathway could not find corresponding unigene (Table S17). In chlorophyll degradation steps, the enzymes NYC1, HCAR, PPH, PAO and RCCR can find one or more corresponding unigene(s), but PPD enzyme in the classic pathway could not find unigene (Table S17). Based on these identified unigenes the proposed chlorophyll metabolic pathway in tepals of Chinese narcissus was constructed (Figure 9).

The expression change trends of the 51 unigenes were compared in tepals from T2 to T5 stages based on their RPKM value. The additive expression levels of the multiple enzyme family genes were recalculated. In ALA, protoporphyrin IX, heme and chlorophyll biosynthesis steps, most of the genes in the main precursor synthesis steps (ALA and protoporphyrin IX) showed decreased expression, but, in heme and chlorophyll biosynthesis steps, the genes *HemH*, *COX10* and *COX15* in heme synthesis sub-pathway showed up-regulated expression, whereas the genes *ChlH*, *ChlD*, *ChlI*, *ChlM*, *ChlE*, *4VCR*, *POR*, *ChlG* and *CAO* in chlorophyll biosynthesis sub-pathway showed constantly decreased expression. Remarkably, *ChlH*, *ChlD*, *ChlI*, *ChlE*, *4VCR*, *POR* and *CAO* genes early showed sharp decreased expression in tepals from stage T2 to T3, and at T4 and T5 stages, most of them further decreased to extremely low level, which suggests that these genes are negatively regulated by one (or some) shared regulator(s). In chlorophyll degradation steps, the unigenes *NYC1*, *PPH*, *PAO* and *RCCR* showed obvious increased expression from T2 to T5 stage, just opposite to the change trends of most of the chlorophyll synthesis associated genes (Figure 9). In contrast, *HCAR* gene that catalyzes 7-hydroxymethyl chlorophyll *a* converting to chlorophyll *a* in chlorophyll cycle showed decreased expression, which was consistent with the decreased chlorophyll content change trend in tepals. These results indicate that the chlorophyll synthesis in tepals was negatively controlled by chlorophyll metabolic pathway on transcription level, not only at the precursor synthesis steps, but also at the chlorophyll formation and degradation steps. To assure the DGE-seq result, expression of 12 representative unigenes were detected by semi-qRT-PCR. The result showed that all genes involving in chlorophyll formation (*HemA*, *HemE*, *ChlH*, *ChlE*, *POR* and *CAO*), heme synthesis (*HemH* and *COX10*), and chlorophyll degradation (*NYC1*, *PPH*, *PAO* and *RCCR*) steps can match to the expression change trends of these genes in DGE-seq (Figure 6C), which confirmed the overall transcription level of chlorophyll metabolic pathway and its ability to produce corresponding amount of chlorophyll metabolites in tepals of Chinese narcissus.

To make clear the relationship of gene differential expression with the content change trend of chlorophyll in tepals, the content of chlorophyll a, chlorophyll b and the total chlorophyll in tepals at T2, T3, T4 and T5 stages were detected. The result showed that chlorophyll a, chlorophyll b and total chlorophyll displayed the same content change trends: all of them are significantly increased from T2 to T3 stage, achieve the peak at T3 stage, and then rapidly decrease to extremely low level at T5 stage (Figure 7C and Table S18). The total chlorophyll content was more than 10-fold higher than flavonoid metabolites (Figure 7A), carotenoid metabolites (Figure 7B) and the total carotenoids (Figure 7C) in tepals at T2, T3 and T4 stages, which suggests that chlorophyll may play a major role in tepals that affects tepal color formation of Chinese narcissus.

## 3. Discussion

Concerning no-genome information organisms, the strategy that uses assembled transcriptome as reference and digital gene expression profiling (DGE) for gene differential expression analysis among samples is commonly reported [25]. This strategy overcomes the dependent of complete genome information for transcriptomic or gene expression study, thus facilitates nearly all biological research, regardless of whether the studied materials have complete, partial or no available genome information. This is especially important for research in model plant species without genome information, such as Chinese narcissus. In this study, by using the above strategy combined with intermediate metabolite analysis method, three pigments associated metabolic pathways (flavonoids, carotenoids and chlorophyll) were drawn up and showed significant variations during tepal pigmentation process. The structure of each pigment metabolic pathway was constructed based on unigenes identified in the assembled tepal transcriptome, and the contribution of each enzyme encoding unigene expression to color pigment creation and content changes in each pathway was analyzed. This work displays the first detailed scene of composition of pigment metabolic pathways in tepal and their correlation with tepal color formation during the whole flower development and pigmentation process of Chinese narcissus, which provides valuable theoretical basis for flower color innovations in future.

### 3.1. Contribution of Flavonoids Metabolic Pathway to White Tepal Color Formation of Chinese Narcissus

Flavonoids are the collective name of a large number of metabolites in flavonoids metabolic pathway [13,43]. Among them, anthocyanins as the major red, purple, and blue pigments gain great attentions [11]. The enzyme structure of classic flavonoids metabolic pathway has been set up for decades [12,13,29,44,45]. In this study, a specialized flavonoids metabolic pathway was constructed in tepals of Chinese narcissus (Figure 5). However, remarkably, this pathway was incomplete in anthocyanins synthesis sub-pathway, and also was down-regulated during the whole tepal pigmentation process (Figure 5), which results in negative contribution to flavonoids synthesis in tepals. The four genes *C4H*, *CHS*, *ANS* and *FLS* must be the most critical genes in this pathway. It was reported that C4H in general phenypropanoid pathway controls the generation of *p*-counaric acid, which is indispensable for the synthesis of all flavonoid derivatives [11,13]. CHS is essential for the generation of chalcone, which acts as a precursor for the synthesis of all anthocyanins and most of the other flavonoid metabolites [46,47]. *ANS* is critical for the synthesis of all three anthocyanidins pelargonidin, cyanidin and delphinidin in the anthocyanins synthesis sub-pathway [12,22]. However, in tepals of Chinese narcissus, the expression of *C4H* decreased sharply during the tepal pigmentation process (Figure 5 and Table S8), which indicates that *p*-counaric acid synthesis in tepals is seriously restricted, especially at the late tepal pigmentation stage. Although five *CHS* unigenes were identified (Table 1), similar to *C4H*, the additive expression level of *CHS* in tepals decreased sharply (Figure 5), which also restricts the synthesis of chalcone. The expression of *ANS* was extremely low in tepals (Figure 5), which means that the synthesis of pelargonidin and cyanidin (without delphinidin synthesis for lack of *F3′5′H* gene) should be seriously restrained. Additionally, besides the negative control of the above three genes, some other genes (*C3H/C3′H*, *4CL*, *CCR*, *CAD*, *HCT*, and *CCoAOMT*) in the competitive sub-pathways and the high expression of *FLS* gene in flavone and flavonol sub-pathway also play negative effect on anthocyanins biosynthesis (Figure 5). Further, some anthocyanidin glycosylation related genes (*3GGT*, *UGT79B1*, *3AT*, *5AT*, and *UGAT*) were deficient (Table S9), which means that, even if the tepals can synthesize minimal anthocyanins, due to lack of effective modification, neither of them could convert to stable anthocyanins in vivo [12,48]. Consistently, these above inferences were confirmed by the detection of flavonoid metabolites in tepals (Figure 7).

Naringenin is a central intermediate of flavonoids metabolic pathway. It can be catalyzed by F3H to synthesize dihydrokaempferol (DHK), and then by F3′H and F3′5′H to generate dihydroquercetin (DHQ) and dihydromyricetin (DHM) through anthocyanin synthetic sub-pathway [12,44,49]. Meanwhile, it can be catalyzed by FNS, F2H and IFS through other flavone and flavonol metabolic sub-pathways to generate apigenin, 2-hydroxyflavanone and 2-hydroxyisoflavanone, respectively [12], but not exist in tepals of Chinese narcissus (Table S9). Remarkably, naringenin can also be catalyzed by FLS to synthesize rutin through the F3H-FLS-F3′H-UF3GT-GT4 sub-pathway [13,50]. In tepals of Chinese narcissus, the expression of *FLS* is higher than genes from other competitive sub-pathways (Figure 5), and the content of rutin was equivalent to naringenin. Notably, both displayed the same change trend during tepal pigmentation process (Figure 7A). This indicates that rutin is the major derivative of naringenin, which suggests that naringenin to rutin sub-pathway is the main branch of flavonoids metabolic pathway in tepals, although the whole flavonoids metabolic pathway was negatively regulated (Figure 5).

**Figure 10:**
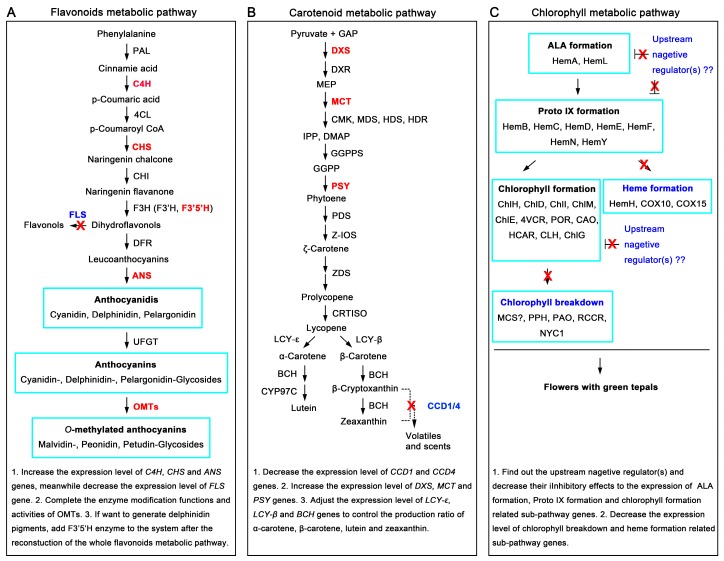
Proposed modification strategies of flavonoids, carotenoids and chlorophyll metabolic pathways in tepals of Chinese narcissus to acquire functional color pigments. (**A**) Reconstruction of flavonoids metabolic pathway to acquire functional anthocyanin pigments (cyanidin, delphinidin, and pelargonodin); (**B**) Modification of expression of some critical genes in carotenoids metabolic pathway to acquire sufficient functional carotenoids pigments; (**C**) Restriction of gene expression in chlorophyll breakdown and heme formation sub-pathways meanwhile increase gene expression in ALA formation, Proto IX formation and chlorophyll formation sub-pathways may produce flower with green tepal. The specific suggestions for modifications of each pathway are listed at the bottom of each section. Black filled arrows indicate the substance metabolism directions; red “X” means the decrease or deletion of the expression of negative gene(s) or sub-pathways in order to remodel the main pathway.

Taken together, analyzing the tepal RNA-seq dataset and detecting the content variation of flavonoid intermediate metabolites indicated that flavonoids metabolic pathway in tepals plays a negative role in regulating flavonoid pigment formation. This may be one of the causes that result in white color formation in tepals of Chinese narcissus. Therefore, if future work were designed to create functional anthocyanins or other flavonoids pigment metabolites, the reconstruction of the flavonoids metabolic pathway in tepals would be necessary (Figure 10).

### 3.2. Contribution of Carotenoids Metabolic Pathway to White Tepal Color Formation of Chinese Narcissus

The classic carotenoids metabolic pathway has been addressed for decades, and the functions of some critical enzymes such as DXS, MCT, PSY and CCDs to affect pigment synthesis in plants have been identified [51,52,53,54,55]. In this study, a specialized carotenoids metabolic pathway was depicted in tepals of Chinese narcissus (Figure 8). The results showed that some genes associated with carotenoid biosynthesis and degradation/cleavage processes have either significant variations or extremely low expression (Figure 8). The additive expression level of *DXS* was lower than 10 fold at T2, T3 and T4 stages than at T5 stage (Figure 8 and Table S12), which suggests that *DXS* initially determines the total carotenoids yield by controlling the supply of precursors (IPP and DMAPP) needed in the carotenoids biosynthesis sub-pathway (Figure 8). Although from T4 to T5 stages the expression of *DXS* was increased and the expression of subsequent genes *DXR*, *HDS*, *HDR*, *GGPPS*, *ZDS* and *CRTISO* were constantly rising, the expression of *MCT* gene in the MEP sub-pathway was always low and *PSY* gene in the carotenoids biosynthesis step was sharply decreased from T3 to T5 stage, the total carotenoids generation rate in tepals should be restricted to low level. This speculation was confirmed by comparing the total carotenoids content in tepals at T2, T3, T4 and T5 stages to the content of chlorophyll (Figure 7C). These results highlight the importance of *DXS*, *MCT* and *PSY* genes in negatively controlling carotenoids synthesis at the precursor synthesis step in tepals, which confirmed their roles that were previously identified in other plants [55,56,57,58,59,60,61]. From detecting the content changes of lutein, β-carotene and zeaxanthin pigments, the result showed that lutein is much higher than β-carotene and zeaxanthin (Figure 7B). This was consistent with the integrated expression effects of *LCY-β*, *LCY-ε*, *BCH* and *CYP97C1* genes that control the synthesis of these substances, and also the *CCD* genes that control the degradation/cleavage of *β-carotene* and its derivatives (zeaxanthin, antheraxanthin and violaxanthin) [32,51,62]. The transcription level of *LCY-ε* was much higher than *LCY-β* during T2 and T3 stage (Figure 8 and Table S12), which means that carotenoids synthesis at lycopene node is more inclined to *α*-carotene sub-pathway than *β-carotene* sub-pathway. Both sub-pathways need BCH enzyme to catalyze the subsequent reactions, and the transcription level of *CYP97C1* was higher than *LCY-β* at T2 and T3 stages (Figure 8 and Table S12), thus the yield rate of lutein in tepals should be higher than *β*-carotene. Additionally, for the expression level of *CCD1* and *CCD4* genes were equivalent to *BCH* (Figure 8), but *BCH* was required for both the *α*- and *β-carotene* sub-pathways, the cleavage rate of *β-carotene* derivatives (*β*-cryptoxanthin and zeaxanthin) catalyzed by CCD1 and CCD4 enzyme should be higher than their yield rate catalyzed by BCH (Figure 8). Therefore, the contents of *β-carotene* and zeaxanthin pigments in *β-carotene* sub-pathway were much lower than lutein pigment in *α*-carotene sub-pathway at last (Figure 7B). The *CCD1/4* genes also play negative role in controlling carotenoids pigment synthesis in tepals.

Taken together, the above analysis revealed that the *DXS*, *MCT*, *PSY* and *CCD1*/*CCD4* genes in carotenoids metabolic pathway play a dominant role in negatively controlling the total carotenoids generation rate and content in tepals of Chinese narcissus (Figure 8). This may be the second cause that results in white color formation in tepals of Chinese narcissus. Therefore, the modification of expression of these genes will help to produce yellow, orange or red color in tepals of Chinese narcissus (Figure 10).

### 3.3. Contribution of Chlorophyll Metabolic Pathway to White Tepal Color Formation of Chinese Narcissus

As a member of tetrapyrroles, chlorophyll is the most abundant pigment in plants [63]. Effects of chlorophyll as a pigment involving in flower color formation has been reported in many flowering plants, such as the variants of chrysanthemum, rose, peony and orchid [15,16,64]. Interestingly, tepals of Chinese narcissus undergo five typical color-change stages that are accompanied with significant chlorophyll synthesis and degradation process (Figure 1).

From analyzing the tepal DGE-seq dataset and content change trends of chlorophyll a, chlorophyll b and the total chlorophyll (Figure 7C and Figure 9), the result showed that chlorophyll metabolism in tepals is more inclined to the degradation side (Figure 9). Although genes in the common ALA (*HemA* and *HemL*) and protoporphyrin IX (*Hem B*, *HemC*, *HemD*, *HemE*, *HemF*, *HemN* and *HemY*) formation steps show nearly parallel expression from T3 to T5 stages, genes in the chlorophyll formation sub-pathway (*ChlH*, *ChlD*, *ChlI*, *ChlM*, *ChlE*, *4VCR*, *POR*, *CAO* and *CLH*) showed decreased expression, while genes in the heme formation sub-pathway (*HemH*, *COX10* and *COX15*) showed significantly elevated expression (Figure 9), the flux of chlorophyll metabolism downstream the protoporphyrin IX node is obviously tilted to the heme formation sub-pathway (Figure 9). Hence, the synthesis of chlorophyll precursor in these steps is suppressed. Further, for most of the chlorophyll degradation associated genes (*NYC1*, *PPH*, *PAO* and *RCCR*) show increased expression from T3 to T5 stage, the total chlorophyll yield in tepals should also be determined in chlorophyll degradation step. Thus, the amount of chlorophyll synthesis in tepals is both negatively controlled by the precursor and chlorophyll degradation steps, which is consistent with the content change trends of chlorophyll a, b and the total chlorophyll in tepals (Figure 7C). This may be the third cause that results in white color formation in tepals of Chinese narcissus. Remarkably, the expression change trends of the enzyme encoded genes in heme formation, chlorophyll formation and chlorophyll breakdown sub-pathways displayed nearly uniform up- or down-regulated manner (Figure 9), which indicates that these genes are likely regulated by the same regulator(s). Thus, the identification of these one or more regulatory factors may greatly facilitate the understanding of chlorophyll metabolism in tepals, meanwhile help to remodel the tepal color formation of Chinese narcissus through the chlorophyll metabolic pathway in future (Figure 10).

### 3.4. Interplay between Tepal Development Stage and Light Exposure on Regulating Flavonoids, Carotenoids and Chlorophyll Metabolisms to Influence Early Tepal Pigmentation Patterns of Chinese Narcissus

As mentioned above, content change trends of metabolites in flavonoids, carotenoids and chlorophyll metabolic pathways from T3 to T5 stages are mostly concerted with the expression change trends of critical enzyme encoded genes (e.g., *C4H*, *CHS*, *ANS*, *FLS*, *DXS, MCT, PSY*, *CCD4*, *POR*, *PPH*, *PAO*) in each pigment associated metabolic pathway. However, from T2 to T3 stage the contents of pigments are raising, but the expression of pigment synthesis associated genes are decreasing meanwhile the degradation/cleavage associated genes are rising (Figure 5, Figure 6, Figure 7 and Figure 8), which displayed conflicts between the pigment synthesis amount and the integrated gene expression level in tepals. This may be explained by the supposition that tepal development stage and light exposure coordinately influence the yield of flavonoids, carotenoids and chlorophyll pigments at the early tepal pigmentation stage (Figure 5, Figure 8 and Figure 9). Thus, the activities of some enzymes in each pigment metabolic pathway are regulated by both the development stage and light, such as the PAL, 4CL, CHS, CHI, DXS, PSY and POR enzymes [65,66,67,68]. This similar situations have also been observed in Arabidopsis seedlings and roots [66,69], barley shoots [70], mustard cotyledons [71], tobacco leaves [72], and grape and bilberry fruits [73,74,75], which indicates a certain universality in angiosperms.

### 3.5. Superposition Effect of Flavonoids, Carotenoids and Chlorophyll Metabolism on the White Tepal Color Formation of Chinese Narcissus

As mentioned in the Introduction, various flower colors in plants are the result of evolution [5,6,7,8,9,10]. Color evolution is consistent with the spectral characteristics of sunlight, that is, green color as the starting point, to extend to long wavelength generates yellow to orange to red color, whereas extend to short wavelength produces blue to purple color [5,10]. The tepals and corona both exhibit green color in Spanish green narcissus (*Narcissus viridiflorus* Schousb.), which is one of the original species of genus “*Narcissus*”. However, with the evolution of narcissus species, new varieties with diverse flower color are continuously coming out, such as *Narcissus trumpet*, *Narcissus large-cupped*, *Narcissus small-cupped*, *Narcissus double*, *Narcissus incomparabilis*, *Narcissus jonguilla*, *Narcissus tazetta*, *Narcissus cyclamineus*, *Narcissus poeticus*, etc. [7,76]. Interestingly, tepals of these narcissus varieties mostly display white or yellow color, whereas corona usually display yellow, orange or red color [18], which indicates that the flower color of narcissus species might have evolved toward long wavelength of light. Therefore, chlorophyll and carotenoids metabolic pathways might be the main pathways in narcissus species to generate visible color pigments. In Chinese narcissus, though chlorophyll, carotenoids and flavonoids derived pigments or intermediates can be detected in tepals, but for chlorophyll content was more than 10-fold higher than other pigments or intermediates (Figure 7), the superposition effect of flavonoids, carotenoids and chlorophyll metabolic pathways on tepal color formation is more likely to be determined by the generation of chlorophyll metabolites, thus chlorophyll metabolism may play the major role in deciding white tepal color formation in Chinese narcissus.. As for why corona color turns yellow in mature flowers (Figure 1), it is speculated that carotenoids metabolic pathway may play the major role that is different from its actions in tepal. To make answer these questions, detailed insights would be made into deciphering the flower color formation mechanism of Chinese narcissus in future work.

## 4. Materials and Methods

### 4.1. Plant Materials

Chinese narcissus cultivated variety “Jingzhanyintai” were purchased from local farmer (Zhangzhou, Fujian, China) in October 2013 and 2014. After removing old bulb scale and roots, cleaned bulbs were sand cultured in a greenhouse set at 23 °C with constant illumination (16 h light/8 h dark) (Figure 1A,B). Tepals were firstly collected at the fifth day (sample T2) as sheathed leaves emerged from bulbs (Figure 1B,C,G,H), then collected at another three stages (samples T3–T5) as umbel in spathe developed (Figure 1D–F,I–N). All samples were immediately frozen in liquid nitrogen and stored at −80 °C for subsequent RNA-seq, DGE-seq, semi-qRT-PCR, and metabolites analysis.

### 4.2. RNA Extraction, RNA-Seq Library Construction, and Sequencing

Total RNA from each sample (sample T2–T5) was extracted using a commercial plant total RNA extraction kit (RP3202, Bioteke Corporation, Beijing, China), quantified on a NanoDrop2000 UV-Vis spectrophotometer (Thermo Scientific, Waltham, MA, USA), and assessed the integrity on an Agilent2100 Bioanalyzer (Agilent, Santa Clara, CA, USA). The qualified RNA with a RIN value higher than 8.0 was utilized to construct the RNA-seq library. To obtain complete tepal transcriptome information, a pooled total RNA sample (Sample Tmix) equally mixed by total RNA from T2 to T5 tepals was used for library construction. Briefly, poly (A)+ RNA was purified from 20 mg of pooled total RNA using oligo (dT) magnetic beads and then broken into short fragments. Taking these cleaved mRNA fragments as templates, first- and second-strand cDNA were synthesized. The resulting cDNAs were purified with the QiaQuick PCR extraction kit (QIAGEN, Hilden, Germany) and resolved with elution buffer for end reparation and for adding poly (A). Thereafter, the short fragments were connected with Illumina paired-end solexa adaptors. After agarose gel electrophoresis, suitable fragments were chosen for PCR amplification as templates. Finally, the library was sequenced on an Illumina HiSeq™ 2500 sequencing system at the Genomic Platform of the Institute for Genome and Biotechnology of Fujian Agriculture and Forestry University. All raw data were deposited in the GenBank Short Read Archive (Accession SRP083093).

### 4.3. DGE-Seq Library Construction and Sequencing

mRNA from T2 to T5 tepal samples were respectively enriched and purified with oligo (dT) magnetic beads according to the Illumina manufacturer’s instruction. After adding the fragmentation buffer, the mRNA was interrupted to short fragments (about 200 bp). Then, the first strand cDNA was synthesized by a random hexamer-primer using the mRNA fragments as templates. The resulting cDNA was purified with a QiaQuick PCR extraction kit (QIAGEN) and resolved with elution buffer for end repair and single nucleotide “A” addition. Finally, sequencing adaptors were ligated to the fragments. Suitable fragments were purified by agarose gel electrophoresis and enriched by PCR amplification. The products were sequenced on an Illumina HiSeq™ 2500 sequencing system at the Genomic Platform of the Institute for Genome and Biotechnology of Fujian Agriculture and Forestry University. The raw data were deposited in the GenBank Short Read Archive (Accession SRP083092).

### 4.4. De Novo Transcriptome Assembly and Annotation

Raw reads were generated by base calling. After judging the sequencing quality (Figure S1) and filtering raw reads, de novo assembly of transcriptome was carried out using the Trinity platform with the parameters of “*K*-mer = 25, *K* = 2, group pairs distance = 500” [25]. Considering the sequencing strategies used in this study, all of the raw data (one RNA-seq and 4 DGE-seq library pools) were used to assemble the tepal transcriptome. The generated unigenes were aligned to a series of protein databases using BLASTx (*E*-value ≤ 10–5) to get an annotation; these databases include the NCBI non-redundant (Nr), Swiss-Prot, COG, KEGG, and GO [77,78,79]. To determine gene coverage, the reference sequences of the classic pigment-related metabolic pathways were downloaded from KEGG and Swiss-Prot. All isoforms of the color-related genes presented in the transcriptome were discovered and aligned against to the corresponding reference sequences using the BLASTx tool to assure the validity of the annotation.

### 4.5. Mapping of DGE Tags and Expression Caculation

Raw data of DGE-seq libraries from T2 to T5 tepals were filtered to remove invalid tags respectively. The remaining clean tags were aligned to the assembled tepal transcriptome using SOAPaligner/soap2 tool [80], then normalized into RPKM values (reads per kb per million reads) by RSEM software [81]. To assure the statistic validity, the reads randomness distribution and saturation status in each library were assessed (Figure S2). After that, the differential expression of unigenes among different stage tepals were compared by using EBSeq [82] with the FDR significance score ≤0.01 and an absolute value of the log_2_ ratio ≥1. In each pigment associated metabolic pathway, some unigenes belong to multiple enzyme gene family (such as the CHS enzyme gene family) that do not display unanimous expression level in the same tepal stage, thus the expression of these unigenes was represented by their additive effect. The results were displayed as heatmaps by using Heatmap Illustrator HemI [83].

### 4.6. Gene Expression Validation

Expression of representative unigenes in each metabolic pathway was confirmed by semi-qRT-PCR (semi-quantitative reverse transcription and polymerase chain reaction) with specific primers designed by Beacon Designer^TM^ (Ver. 7, Palo Alto, CA, USA) (Table S19). cDNA synthesis was done by using RevertAid First Strand cDNA Synthesis Kit (Thermo Scientific, Waltham, MA, USA) following the manufacturer’s protocol. Semi-qRT-PCR was performed on an ARKTIK thermal cycler (Thermo Scientific). The unigene annotated to *GAPDH* (*c28660.graph_c0*) was chosen as an internal control (25 cycles). Three independent biological repetitions with three technical replicates were performed. PCR products were run on a 2.0% TAE agarose gel and calculated by measuring the grey level of each lane using AlphaView software (San Jose, CA, USA).

### 4.7. Measurement of Contents of Flavonoids and Carotenoids Metabolites in Tepals by HPLC

For extraction of flavonoids metabolites for HPLC analysis, a method described by Schieber et al. was adopted with minor modifications [84]. Briefly, freeze-dried tepals were finely grounded in liquid nitrogen and 100 mg was extracted in 1 mL of MeOH/DMSO mix (1:1, *v*/*v*) for 30 min at 4 °C with agitation (190 rpm) in darkness. Then samples were centrifuged at 4 °C, 9000 rpm for 15 min, the supernatants were transferred to fresh tubes and the pellets were re-extracted for another 4 times until the color changed into white. All the supernatants were combined and filtered through a 0.22 μm membrane filter. The HPLC was carried out according to a previous method on a Agilent 1260 HPLC system [85]. All the flavonoids standards including naringenin, eriodictyol, dihydroquercetin, kaempferol, quercetin, rutin and caffeic acid (all purchased from Sigma-Aldrich，St. Louis, MI, USA) were prepared in MeOH/DMSO (1:1, *v*/*v*) and stored at −20 °C before use. For extraction of carotenoids, the method described by Pintea et al. [86] was referenced with some modifications. Briefly, 100 mg finely grounded tepal powder was instantly mixed with 1 mL of methanol/ethyl acetate/petroleum ether mix (1:1:1, *v*/*v*/*v*) containing 0.004% butylated hydroxytoluene (BTH) as antioxidant and calcium carbonate, then kept at 4 °C with agitation (190 rpm) in darkness for 30 min. The samples were centrifuged at 4 °C, 10,000 rpm for 6 min, the supernatants were then transferred to fresh tubes and the pellets were re-extracted for another 3 times. For removal of soaps and alkalies, the solution was washed 2 times with a sodium chloride-saturated solution and distilled water, respectively. The organic layer containing carotenoids was dried over in a rotary vacuum evaporator at 30 °C, then the concentrate was dissolved with 0.5 mL of methyl tert-butyl ether (MTBE, containing 0.01% BTH) and filtered through a 0.22 μm membrane filter. The HPLC was performed according to YMC separation technology-Carotene and xanthophylls protocol with minor modifications on the Agilent 1260 HPLC system (Agilent, Santa Clara, CA, USA). Carotenoid standards including lutein, β-carotene, zeaxanthin and astaxanthin were bought from Sigma-Aldrich. BHT in MTBE (0.01%) was added to all of the standards, and they were diluted with MTBE (containing 0.01% BHT) before use. To reduce the measurement error, the reference standard of every flavonoid (or carotenoid) metabolite was prepared stock solution respectively and mixed together in a diluted mixture under appropriate concentrations, and the chromatographic condition for fine separability of each metabolite in the mixture was adjusted. After that, the composition of these flavonoid compounds (or carotenoid compounds) and their contents in every pigmentation stage of tepals were studied for three biological repetitions with three technical replicates.

### 4.8. Measurement of Chlorophyll and the Total Carotenoids Contents in Tepals

From 50 to 100 mg fresh weight of tepals from T2 to T5 stages were harvested. Pigments were extracted by immersing tepals in 1 mL of 95% ethanol (*v*/*v*) and kept at 4 °C in dark for 48 h until the tissues became white. The extract was used to measure the absorbance at 470, 649, and 665 nm in 96-holes ELISA plate by using a Flexstation 3 Microplate Reader (Berthold, Bad Wildbad, Germany). Each hole was filled with 200 μL extract and pure 95% ethanol was used as a control. Chlorophyll a, b, the total chlorophyll and the total carotenoids contents were calculated according to the method described by Wellburn [87]. All detections were done for at least three biological repetitions with three technical replicates.

### 4.9. Statistical Analyses

Mean values and standard error (SE) were calculated in this research by using Microsoft Office Excel 2007. Significant differences between samples were made by software Origin 7.5 using the student’s *t*-tests. ** represents *p* < 0.01,* represents *p* < 0.05.

## 5. Conclusions

In this study, by using genome-wide transcriptome profiling and metabolites analysis methods, we set up the tepal transcriptome of Chinese narcissus, constructed three metabolic pathways of flavonoids, carotenoids, and chlorophyll, and dissected the contributions of these pathways to the final tepal color formation of the wildly cultivated narcissus variety “Jingzhanyintai”. The result showed that all these three pathways exhibit negative control of color pigments synthesis in tepals, finally resulting in the formation of white tepals. And among then, the chlorophyll metabolic pathway may play the major role in deciding tepal color formation. To our knowledge, this is the first research focusing on the whole genomic molecular analysis of flower pigmentation patterns in narcissus species. This work provides valuable theoretical basis for Chinese narcissus tepal color formation and genomic resources for candidate genes applicable for flower color innovations through the flavonoids, carotenoids and chlorophyll metabolic pathways in future.

## Figures and Tables

**Table 1:** Unigenes related to flavonoids metabolic pathway in tepals of Chinese narcissus.

Function	Gene	Enzyme	KO ID (EC No.)	No. All *^a^*
Phenylpropanoid biosynthesis	*PAL*	Phenylalanine ammonia-lyase	K10775 (4.3.1.24)	4
	*C4H*	Cinnamate 4-hydroxylase	K00487 (1.14.13.11)	1
	*4CL*	4-Coumarate:CoA ligase	K01904 (6.2.1.12)	12
	*C3H*	p-Coumarate 3-hydroxylase	\*^b^* (1.14.13.-)	1
	*C3′H*	Coumaroylquinate (coumaroylshikimate) 3′-monooxygenase	K09754 (1.14.13.36)	
	*COMT*	Caffeic acid 3-*o*-methyltransferase	K13066 (2.1.1.68)	3
	*F5H*	Ferulate-5-hydroxylase	K09755 (1.14.-.-)	1
	*CCR*	Cinnamoyl-CoA reductase	K09753 (1.2.1.44)	13
	*CAD*	Cinnamyl-alcohol dehydrogenase	K00083 (1.1.1.195)	12
	*HCT*	Shikimate *o*-hydroxycinnamoyltransferase	K13065 (2.3.1.133)	6
	*CCoAOMT*	Caffeoyl-CoA *o*-methyltransferase	K00588 (2.1.1.104)	4
Flavonoid biosynthesis	*CHS*	Chalcone synthase	K00660 (2.3.1.74)	5
	*CHI*	Chalcone isomerase	K01859 (5.5.1.6)	2
	*F3H*	Flavanone 3-hydroxylase	K00475 (1.14.11.9)	3
	*F3′H*	Flavonoid 3′-hydroxylase	K05280 (1.14.13.21)	2
	*DFR*	Dihydroflavonol 4-reductase	K13082 (1.1.1.219)	2
	*ANS*	Anthocyanidin synthase	K05277 (1.14.11.19)	1
Anthocyanin modification	*UFGT*	UDP-glucose:anthocyanidin 3-*o*-glucosyltransferase	K12930 (2.4.1.115)	10
	*3RT*	Anthocyanidin 3-glucoside rhamnosyltransferase	\*^b^* (2.4.1.159)	1
	*5GT*	Cyanidin 3-*o*-rutinoside 5-*o*-glucosyltransferase	\*^b^* (2.4.1.116)	3
	*AA7GT*	Cyanidin 3-*o*-glucoside 7-*o*-glucosyltransferase	K17192 (2.4.1.300)	1
	*5MaT1*	Anthocyanin 5-*o*-glucoside-6′′′-*o*-malonyltransferase	K12934 (2.3.1.172)	2
	*UGT75C1*	Anthocyanin 5-*o*-glucosyltransferase	K12338 (2.4.1.298)	2
	*GT1*	Anthocyanidin 5,3-*o*-glucosyltransferase	K12938 (2.4.1.-)	4
	*3′GT*	Anthocyanin 3′-*o*-β-glucosyltransferase	K12939 (2.4.1.238)	5
Flavone and flavonol biosynthesis	*FLS*	Flavonol synthase	K05278 (1.14.11.23)	4
	*LuOMT*	Luteolin *o*-methyltransferase	\*^b^* (2.1.1.75)	1
	*SOT*	Flavonol 3-sulfotransferase	K13270 (2.8.2.25)	3
	*F4ST*	Flavonol 4′-sulfotransferase	K13271 (2.8.2.27)	1
	*GT4*	UDP-rhamnose:rhamnosyltransferase	\*^b^* (2.4.1.159)	3
	*UF3GT*	Flavonol 3-*o*-glucosyltransferase	K10757 (2.4.1.91)	1
Flavanone biosynthesis	*ANR*	Anthocyanidin reductase	K08695 (1.3.1.77)	1
	*LAR*	Leucoanthocyanidin reductase	K13081 (1.17.1.3)	1

*^a^* No. All, the total number of unigenes found in the annotated tepal transcriptome; \*^b^*, Omission of number for the KO ID.

**Table 2:** Unigenes related to carotenoids metabolic pathway in tepals of Chinese narcissus.

Function	Gene	Enzyme	KO ID (EC No.)	No. All *^a^*
MEP pathway	*DXS*	1-Deoxy-d-xylulose-5-phosphate synthase	K01662 (2.2.1.7)	5
	*DXR*	1-Deoxy-d-xylulose 5-phosphate reductoisomerase	K00099 (1.1.1.267)	1
	*MCT*	2-*C*-methyl-d-erythritol 4-phosphate cytidylyltransferase	K00991 (2.7.7.60)	1
	*CMK*	4-(cytidine 5′-diphospho)-2-*c*-methyl-d-erythritol kinase	K00919 (2.7.1.148)	1
	*MDS*	2-*C*-methyl-d-erythritol 2,4-cyclodiphosphate synthase	K01770 (4.6.1.12)	1
	*HDS*	4-Hydroxy-3-methylbut-2-en-1-yl diphosphate synthase	K03526 (1.17.7.1)	2
	*HDR*	4-Hydroxy-3-methylbut-2-enyl diphosphate reductase	K03527 (1.17.7.4)	1
	*IPPI*	Isopentenyl diphosphate isomerase	K01823 (5.3.3.2)	2
Carotenoid biosynthesis	*GGPPS*	Geranylgeranyl pyrophosphate synthase	K13789 (2.5.1.29)	4
	*PSY*	Phytoene synthase	K02291 (2.5.1.32)	4
	*PDS*	Phytoene desaturase	K02293 (1.3.5.5)	2
	*Z-ISO*	zeta-Carotene isomerase	K15744 (5.2.1.12)	1
	*ZDS*	zeta-Carotene desaturase	K00514 (1.3.5.6)	1
	*CRTISO*	Carotenoid isomerase	K09835 (5.2.1.13)	6
	*LCY-ε*	Lycopene ε-cyclase	K06444 (5.5.1.18)	1
	*LCY-β*	Lycopene β-cyclase	K06443 (5.5.1.19)	1
	*CYP97A3*	Carotene β-hydroxylase (cytochrome P450 type)	K15747 (1.14.-.-)	1
	*CYP97C1*	Carotene ε-hydroxylase (cytochrome P450 type)	K09837 (1.14.99.45)	1
	*BCH*	β-carotene hydroxylase (non-heme di-iron type)	K15746 (1.14.13.129)	2
	*ZEP*	Zeaxanthin epoxidase	K09838 (1.14.13.90)	4
	*VDE*	Violaxanthin de-epoxidase	K09839 (1.23.5.1)	2
	*CCS*	Capsanthin/capsorubin synthase	K14593 (5.3.99.8)	1
Carotenoid degradation	*CCD1*	Carotenoid-9′,10′-cleaving dioxygenase	K10252 (1.13.11.71)	3
	*CCD4*	8′-apo-β-Carotenoid 14′,13′-cleaving dioxygenase	\*^b^* (1.13.11.67)	2
	*CCD7*	9-*cis*-β-carotene 9′, 10′-cleaving dioxygenase	K17912 (1.13.11.68)	1
	*CCD8*	all-trans-10′-apo-β-Carotenal 13,14-cleaving dioxygenase	K17913 (1.13.11.70)	1
	*NCED1*	9-*cis*-Epoxycarotenoid dioxygenase	K09840 (1.13.11.51)	1
	*AAO3*	Abscisic-aldehyde oxidase	K09842 (1.2.3.14)	1
	*CYP707A*	(+)-Abscisic acid 8′-hydroxylase	K09843 (1.14.13.93)	5
	*BG1*	β-d-glucopyranosyl abscisate β-glucosidase	K15748 (3.2.1.175)	2

*^a^* No. All, the total number of unigenes found in the annotated tepal transcriptome; \*^b^*, Omission of number for KO ID.

**Table 3:** Unigenes related to chlorophyll metabolic pathway in tepals of Chinese narcissus.

Function	Gene	Enzyme	KO ID (EC No.)	No. All *^a^*
ALA formation	*HemA*	Glutamyl-tRNA reductase	K02492 (1.2.1.70)	3
	*HemL*	Glutamate-1-semialdehyde 2,1-aminomutase	K01845 (5.4.3.8)	2
Proto IX formation	*HemB*	Porphobilinogen synthase	K01698 (4.2.1.24)	1
	*HemC*	Hydroxymethylbilane synthase	K01749 (2.5.1.61)	2
	*HemD*	Uroporphyrinogen-III synthase	K01719 (4.2.1.75)	1
	*HemE*	Uroporphyrinogen decarboxylase	K01599 (4.1.1.37)	2
	*HemF*	Coproporphyrinogen-III oxidase	K02495 (1.3.99.22)	2
	*HemN*	Oxygen-independent coproporphyrinogen-III oxidase	K02495 (1.3.99.22)	1
	*HemY*	Oxygen-dependent protoporphyrinogen oxidase	K00231 (1.3.3.4)	2
Heme formation	*HemH*	Ferrochelatase	K01772 (4.99.1.1)	4
	*COX10*	Protoheme IX farnesyltransferase	K02257 (2.5.1.-)	1
	*COX15*	Cytochrome c oxidase assembly protein subunit 15	K02259 (1.9.3.1)	2
Chlorophyll formation	*ChlH*	Magnesium chelatase subunit H	K03403 (6.6.1.1)	2
	*ChlD*	Magnesium chelatase subunit D	K03404 (6.6.1.1)	1
	*ChlI*	Magnesium chelatase subunit I	K03405 (6.6.1.1)	4
	*ChlM*	Magnesium protoporphyrin IX methyltransferase	K03428 (2.1.1.11)	1
	*ChlE*	Magnesium-protoporphyrin IX monomethyl ester (oxidative) cyclase	K04035 (1.14.13.81)	1
	*4VCR*	Divinyl chlorophyllide a 8-vinyl-reductase	K19073 (1.3.1.75)	1
	*POR*	Protochlorophyllide reductase	K00218 (1.3.1.33)	5
	*CAO*	Chlorophyllide a oxygenase	K13600 (1.14.13.122)	1
	*ChlG*	Chlorophyll synthase	K04040 (2.5.1.62)	1
	*CLH*	Chlorophyllase	K08099 (3.1.1.14)	3
Chlorophyll degradation	*NYC1*	Chlorophyll (ide) b reductase	K13606 (1.1.1.294)	4
	*HCAR*	7-Hydroxymethyl chlorophyll a reductase	K18010 (1.17.7.2)	1
	*PPH*	Pheophytinase	\*^b^* (3.1.1.-)	1
	*PAO*	Pheophorbide a oxygenase	K13071 (1.14.15.17)	1
	*RCCR*	Red chlorophyll catabolite reductase	K13545 (1.3.7.12)	1

*^a^* No. All, the total number of unigenes found in the annotated tepal transcriptome; \*^b^*, Omission of number for KO ID.

## Supplementary Materials

Supplementary materials can be found at www.mdpi.com/1422-0067/18/9/1923/s1.
