# Supplementary material for: Structure of Pigment Metabolic Pathways and Their Contributions to White Tepal Color Formation of Chinese Narcissus tazetta var. chinensis cv Jinzhanyintai
Source: Int J Mol Sci. 2017 Sep 8;18(9):1923. doi: 10.3390/ijms18091923 (PMC5618572; doi:10.3390/ijms18091923)
Supplement: Supplementary file 1 [file ijms-18-01923-s001.zip › ijms-220392-supplementary - for final.pdf]

Supplementary Material

Structure of Pigment Metabolic Pathways and their Contribution in Controlling Tepal Color Formation of Chinese *Narcissus tazetta* during Flower Development and Pigmentation Processes

Ren Yujun †, Yang Jingwen †, Lu Bingguo, Jiang Yaping, Chen Haiyang, Hong Yuwei, Binghua Wu, Miao Ying *

Correspondence: ymiao@fafu.edu.cn

1 Supplementary Tables

Table S1. Summary of RNA-seq data of tepal transcriptome of Chinese *Narcissus tazetta*.

SampleID	BaseSum (nt)	ReadSum	AveRead (nt)	GC (%)	Q20 (%)	Q30 (%)
Tmix	7,006,263,546	34,684,473	101	48.97	95.62	89.65
T2	1,235,824,486	6,117,943	101	49.37	95.47	89.53
T3	1,368,280,936	6,773,668	101	49.16	95.62	89.74
T4	1,279,109,652	6,332,226	101	49.10	95.54	89.70
T5	1,143,256,572	5,659,686	101	49.20	95.52	89.54
Total	12,032,735,192	59,567,996				

Table S2. Summary of sequence assembly of tepal transcriptome of Chinese *Narcissus tazetta*.

Length range	Contigs	Transcripts	Unigenes
200 -300	3,251,621 (98.45%)	29,915 (22.16%)	24,048 (38.09%)
300-500	23,045 (0.70%)	24,750 (18.34%)	15,718 (24.89%)
500-1000	14,450 (0.44%)	28,313 (20.97%)	10,290 (16.30%)
1000-2000	9,298 (0.28%)	34,342 (25.44%)	8,541 (13.53%)
>2000	4,285 (0.13%)	17,665 (13.09%)	4,544 (7.20%)
Total number	3,302,699	134,985	63,141
Total length (bp)	210,805,969	137,475,863	44,423,386
N50 length (bp)	49	1,619	1,261
Mean length (bp)	64	1018	704

Table S3. Assessment of RNA-seq quality compared with assembled tepal transcriptome.

Samples	ReadSum	Total mapping	Uniquely-mapping	Multiply-mapping
Tmix	34,684,473	28,696,106 (82.73%)	14,107,668 (49.16%)	14,588,438 (50.84%)
T2	6,117,943	5,059,827 (82.70%)	2,508,883 (49.58%)	2,550,944 (50.42%)
T3	6,773,668	5,612,674 (82.86%)	2,761,020 (49.19%)	2,851,654 (50.81%)
T4	6,332,226	5,250,680 (82.92%)	2,630,885 (50.11%)	2,619,795 (49.89%)
T5	5,659,686	4,720,887 (83.41%)	2,412,274 (51.10%)	2,308,613 (48.90%)

Table S4. Summary of function annotation of tepal transcriptome of Chinese *Narcissus tazetta*.

**Annotation Database**	**Annotated number**	**300≤length<1000**	**length≥1000**
COG annotation	8977	2519	5398
GO annotation	21368	7782	9566
KEGG annotation	6801	2357	3165
SwissProt annotation	22325	7904	10905
NR annotation	29598	11591	12402
Total annotated transcripts	29730	11655	12415

Table S5. KEGG pathway mapping of unigenes in tepal transcriptome of Chinese *Narcissus tazetta*.
(Please refer to the Excel file **Table S5** for detailed information).

Table S6. Global and overview mapping of unigenes in KEGG pathways.
(Please refer to the Excel file **Table S6** for detailed information).

Table S7. CDS and protein sequences of flavonoids metabolic pathway associated unigenes in tepal transcriptome of Chinese *Narcissus tazetta*.
(Please refer to the Excel file **Table S7** for detailed information).

Table S8. List of unigenes in flavonoids metabolic pathway in tepal transcriptome of Chinese *Narcissus tazetta*.
(Please refer to the Excel file **Table S8** for detailed information).

Table S9. Enzymes in flavonoids metabolic pathway that cannot find corresponding unigenes in tepal transcriptome of Chinese *Narcissus tazetta*.

**Function**	**Gene**	**Enzyme**	**KO ID (EC No.)**
Phenylpropanoid biosynthesis	C2H	Cinnamic 2-hydroxylase	\ [a] (1.14.13.14)
	FDC1	Phenacrylate decarboxylase	K20039 (4.1.1.102)
Flavonoid biosynthesis	***F3′5′H***	Flavonoid 3',5'-hydroxylase	K13083 (1.14.13.88)
Anthocyanin modification	*Mt1/Mt2*	Anthocyanin 3'-methyltransferase	K05279 (2.1.1.76)
	Mf1/Mf2	Anthocyanin 3',5'-methyltransferase	\ [a] (2.1.1.-)
	3MaT1	Anthocyanin 3-O-glucoside-6''-O-malonyltransferase	K12931 (2.3.1.171)
	3MaT2	Anthocyanidin 3-O-glucoside-3'',6''-O-dimalonyltransferase	K12932 (2.3.1.-)
	3GGT	Anthocyanidin 3-O-glucoside 2''-O-glucosyltransferase	K12933 (2.4.1.297)
	UGT79B1	Anthocyanidin 3-O-glucoside 2'''-O-xylosyltransferase	K17193 (2.4.2.51)
	3AT	Anthocyanidin 3-O-glucoside 6''-O-acyltransferase	\ [a] (2.3.1.215)

	5MaT2	Anthocyanin 5-O-glucoside-4'''-O-malonyltransferase	K12935 (2.3.1.214)
	5AT	Anthocyanin 5-aromatic acyltransferase	K12936 (2.3.1.153)
	UGAT	Cyanidin 3-O-glucoside 2''-O-glucuronosyltransferase	K12937 (2.4.1.254)
	UA3′5′GZ	Delphinidin 3',5'-O-glucosyltransferase	\[a] (2.4.1.249)
Flavone and flavonol biosynthesis	***FNS***	Flavone synthase	K13077 (1.14.11.22)
	C12RT1	Flavanone 7-O-glucoside 2''-O-beta-L-rhamnosyltransferase	K13080 (2.4.1.236)
	FOMT	Flavonol 3-O-methyltransferase	K05279 (2.1.1.76)
	CROMT2	Flavonoid O-methyltransferase	K13272 (2.1.1.267)
	GUSB	beta-Glucuronidase	K01195 (3.2.1.31)
Aurone biosynthesis	***AS1***	Aureusidin synthase	K13079 (1.21.3.6)
Isoflavonoid biosynthesis	***IFS***	2-Hydroxyisoflavanone synthase	K13257 (1.14.13.136)
	HIDH	2-Hydroxyisoflavanone dehydratase	K13258 (4.2.1.105)

\[a], Omission of number for the KO ID.

Table S10. Contents of flavonoid metabolites in tepals of Chinese *Narcissus tazetta* at different tepal pigmentation stages.

Stage	DHQ	RT	NG	QC	KF	CA	ED
	Mean ± SD (mg/g FW)						
T2	n.d.	2.0402 ± 0.0877	1.3657 ± 0.0582	n.d.	n.d.	n.d.	n.d.
T3	n.d.	4.0918 ± 0.0873 **	4.7034 ± 0.1082 **	n.d.	n.d.	n.d.	n.d.
T4	n.d.	2.7622 ± 0.0755 **	3.7545 ± 0.1166 **	n.d.	n.d.	n.d.	n.d.
T5	n.d.	1.5995 ± 0.0564 **	2.1642 ± 0.0756 **	n.d.	n.d.	n.d.	n.d.

Note: n.d. means not detected. The value is averaged from three independent biological experiments with three technical repetitions. DHQ, dihydroquercetin; SD, standard deviation. RT, rutin; NG, Naringenin; QC, quercetin; KF, kaempferol; CA, caffeic acid; ED, eriodictyol; FW, fresh weight. Significant differences between T2 and the other stages were made by software Origin 7.5 using the student's t-tests. ** represents $p < 0.01$.

Table S11. CDS and protein sequences of carotenoids metabolic pathway associated unigenes in tepal transcriptome of Chinese *Narcissus tazetta*.
(Please refer to the Excel file **Table S11** for detailed information).

Table S12. List of unigenes in carotenoids metabolic pathway in tepal transcriptome of Chinese *Narcissus tazetta*.
(Please refer to the Excel file **Table S12** for detailed information).

Table S13. Enzymes in carotenoids biosynthetic pathway that cannot find corresponding unigenes in tepal transcriptome of Chinese *Narcissus tazetta*.

**Function**	**Gene**	**Enzyme**	**KO ID (EC No.)**
Carotenoid degradation	***NSY***	Neoxanthin synthase	K14594 (5.3.99.9)
	CrtO	beta-Carotene ketolase (CrtO type)	K09847 (1.14.15.9)
	CrtW	beta-Carotene ketolase (CrtW type)	K09847 (1.14.15.9)
	ABA2	Xanthoxin dehydrogenase	K09841 (1.1.1.288)
	AOG	Abscisate beta-glucosyltransferase	K14595 (2.4.1.263)

Table S14. Contents of carotenoid metabolites in tepals of Chinese *Narcissus tazetta* at different pigmentation stages.

Stage	Lutein	Zeaxanthin	β-Carotene	Astaxanthin
	Mean ± SD (mg/g FW)			
T2	0.0500 ± 0.0002	0.0024 ± 0.0001	0.0144 ± 0.0003	n.d.
T3	0.1135 ± 0.0062**	0.0105 ± 0.0005**	0.0360 ± 0.0035**	n.d.
T4	0.0513 ± 0.0006	0.0085 ± 0.0002**	0.0121 ± 0.0001	n.d.
T5	0.0042 ± 0.0002**	0.0011 ± 0.0001**	0.0007 ± 0.0001**	n.d.

Note: n.d. means not detected. The value is averaged from three independent biological experiments with three technical repetitions. SD, standard deviation. FW, fresh weight. Significant differences between T2 and the other stages were made by software Origin 7.5 using the student's *t*-tests. ** represents $p < 0.01$.

Table S15. CDS and protein sequences of chlorophyll metabolic pathway associated unigenes in tepal transcriptome of Chinese *Narcissus tazetta*.

(Please refer to the Excel file **Table S15** for detailed information).

Table S16. List of unigenes in chlorophyll metabolic pathway in tepal transcriptome of Chinese *Narcissus tazetta*.

(Please refer to the Excel file **Table S16** for detailed information).

Table S17. Enzymes in chlorophyll metabolic pathway that cannot find corresponding unigenes in tepal transcriptome of Chinese Narcissus tazetta.
\ [a], Omission of number for the KO id.

Table S18. Contents of chlorophyll metabolites and total carotenoids in tepals of Chinese *Narcissus tazetta* at different pigmentation stages.

Stage	Chlorophyll *a*	Chlorophyll *b*	Total chlorophyll	Total carotenoids
	Mean ± SD (mg/g FW)			
T2	40.87 ± 2.1754	29.88 ± 2.3739	70.76 ± 4.3428	8.92 ± 0.5334
T3	136.33 ± 10.0650**	70.73 ± 5.8019**	207.06 ± 15.2406**	27.41 ± 2.4864**
T4	38.71 ± 7.2271	19.19 ± 3.0579**	57.90 ± 10.2549*	8.43 ± 0.9895
T5	1.92 ± 0.1393**	1.82 ± 0.3211**	3.74 ± 0.4497**	1.39 ± 0.1047**

**Function**	**Gene**	**Enzyme**	**KO id (EC no.)**
Proto IX formation	*HemG*	Menaquinone-dependent protoporphyrinogen oxidase	K00231 (1.3.3.4)
Chlorophyll formation	*BchE*	Anaerobic magnesium-protoporphyrin IX monomethyl ester cyclase	K04035 (1.14.13.81)
	BchJ	Divinyl protochlorophyllide a 8-vinyl-reductase	\ [a] (1.3.1.33)
	ChlL	Light-independent protochlorophyllide reductase subunit L	K04037 (1.3.7.7)
	ChlN	Light-independent protochlorophyllide reductase subunit N	K04038 (1.3.7.7)
	ChlB	Light-independent protochlorophyllide reductase subunit B	K04039 (1.3.7.7)
Chlorophyll degradation	*PPD*	Pheophorbidase	K13544 (3.1.1.82)

Note: The value is averaged from three independent biological experiments with three technical repetitions. SD, standard deviation. FW, fresh weight. Significant differences between T2 and the other stages were made by software Origin 7.5 using the student's t-tests. ** represents $p < 0.01$,* represents $p < 0.05$.

Table S19. Primers used for semi-qRT-PCR analysis in this research.
(Please refer to the Excel file **Table S19** for detailed information).

Supplementary Figures

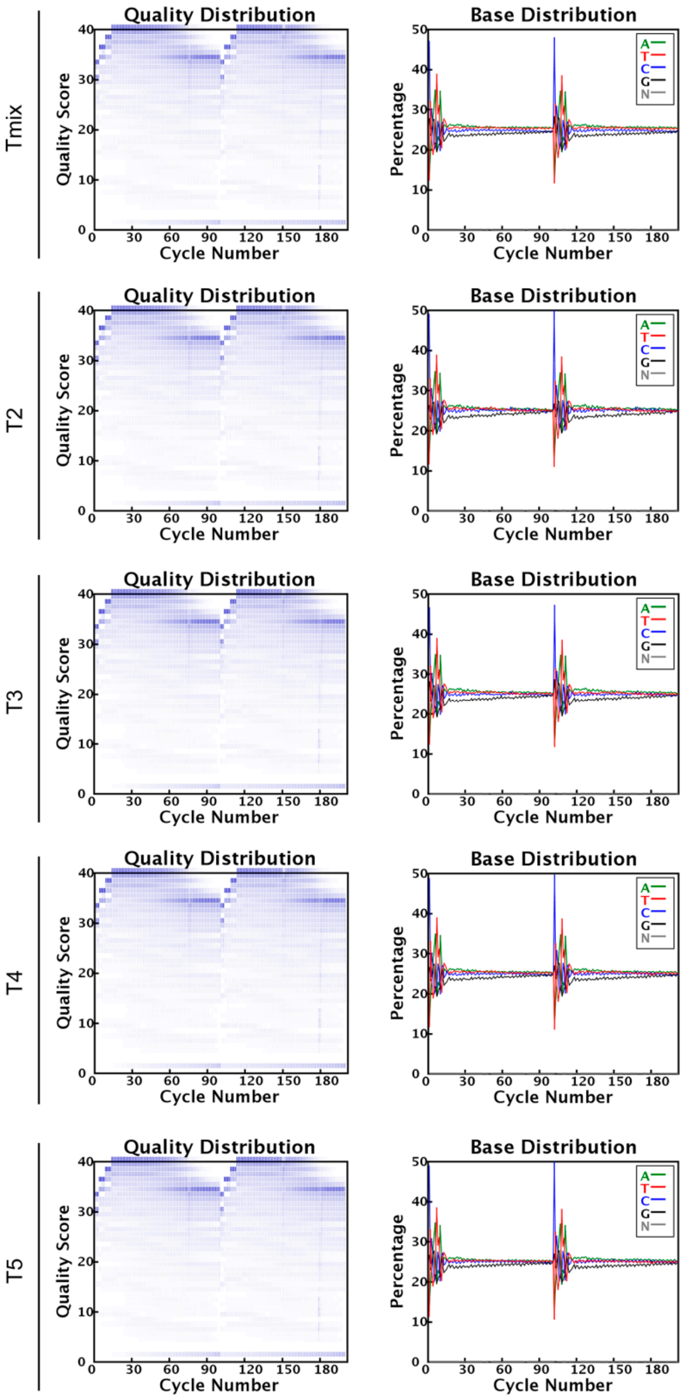

Figure S1. Raw data quality assessment of RNA-seq (Tmix) and DGE libraries based on base error rate (quality distribution) and randomness distribution calculations.

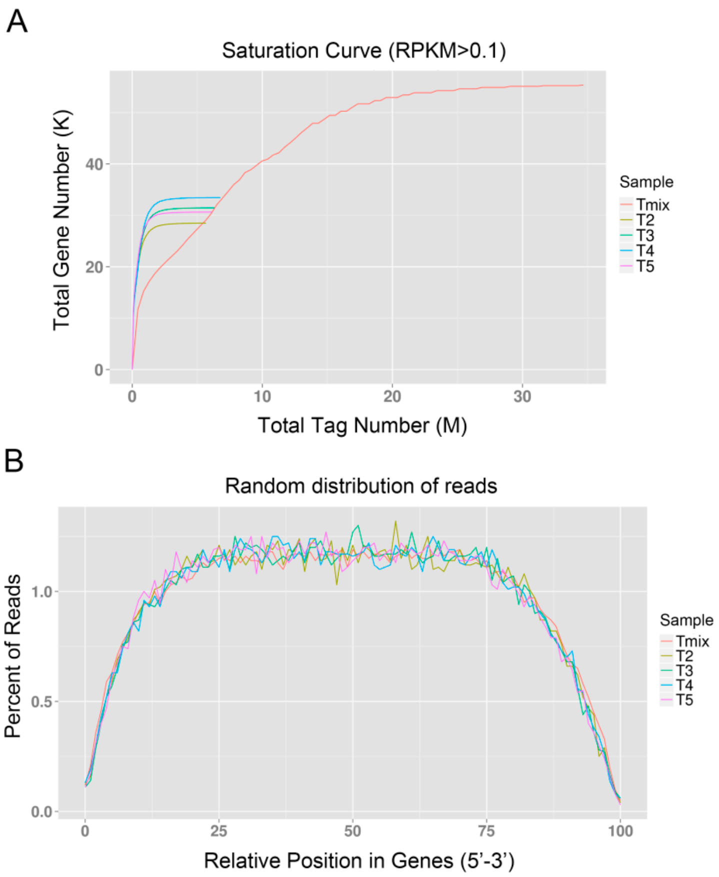

Figure S2. Expression saturation evaluation of sequenced libraries and randomness distribution of reads in assembled unigenes.

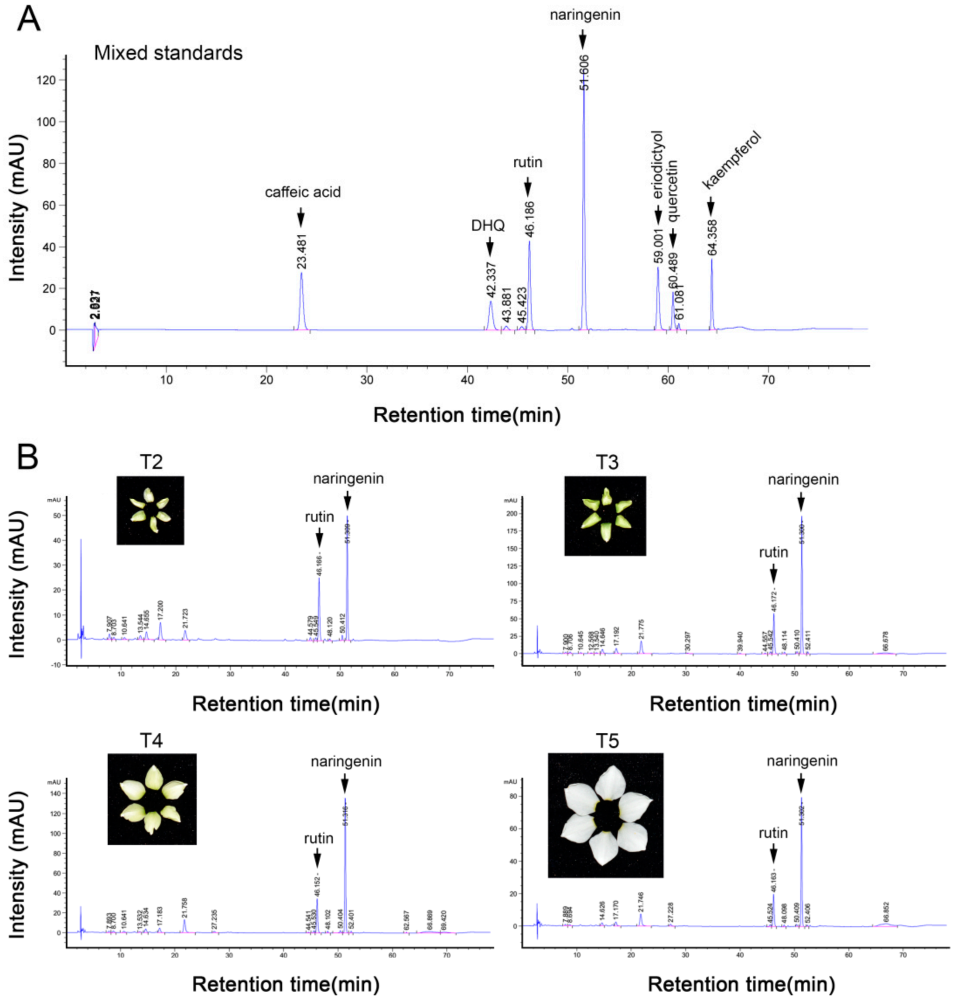

Figure S3. Detection of seven intermediate metabolites of flavonoids metabolic pathway in tepals of Chinese *Narcissus tazetta* at different tepal pigmentation stages by HPLC: (**A**) chromatograms of seven flavonoids standards; and (**B**) chromatograms of flavonoids in tepals at different pigmentation stages.

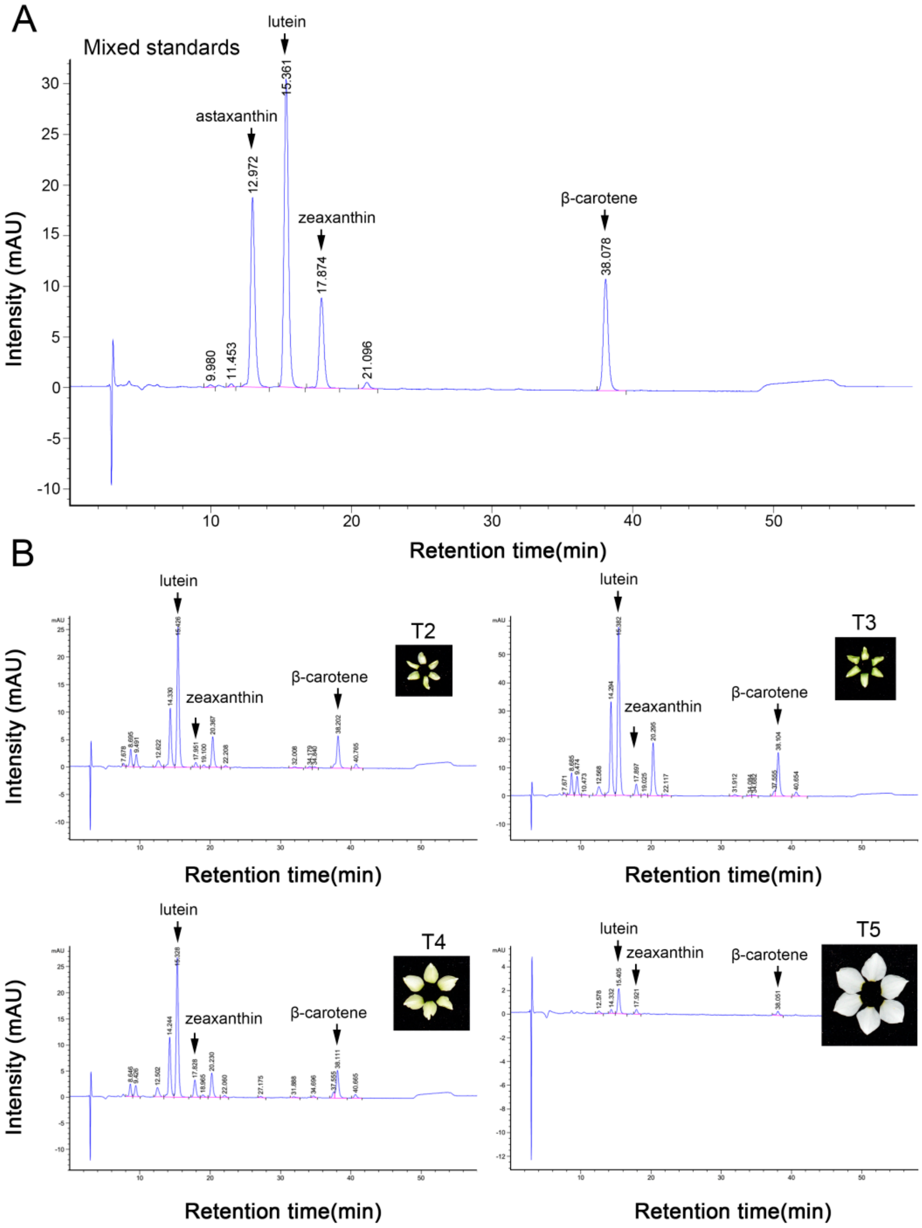

Figure S4. Detection of four intermediate metabolites of carotenoids metabolic pathway in tepals of Chinese Narcissus tazetta at different tepal pigmentation stages by HPLC: (**A**) chromatograms of four carotenoids standards; and (**B**) chromatograms of carotenoids in tepals at different pigmentation stages.
